# Synthesis of Thieno[2,3‐*c*]pyridine Derivatives by 1,2,3‐Triazole‐Mediated Metal‐Free Denitrogenative Transformation Reaction

**DOI:** 10.1002/open.202500060

**Published:** 2025-03-15

**Authors:** Kumsal Eroğlu, Ömer Tahir Günkara, Wim Dehaen

**Affiliations:** ^1^ Department of Chemistry, KU Leuven Sustainable Chemistry for Metals and Molecules Celestijnenlaan 200F B-3001 Leuven Belgium; ^2^ Department of Chemistry Yıldız Technical University Faculty of Art and Science Davutpasa Esenler 34220 Istanbul Türkiye

**Keywords:** 1,2,3-triazoles, metal-free denitrogenative transformation, thieno[2,3-*c*]pyridine

## Abstract

In this study, we report the synthesis of thieno[2,3‐*c*]pyridine derivatives by a metal‐free method via fused 1,2,3‐triazoles. In the synthesis of this skeleton using a three‐step method, we first obtained 1‐(2,2‐dimethoxyethyl)‐5‐(thiophen‐2‐yl)‐1*H*‐1,2,3‐triazole through a one‐pot triazolation reaction and in the next step, we synthesized the thieno[2,3‐*c*][1,2,3]triazolo[1,5‐*ɑ*]pyridine compound using a modified Pomeranz‐Fritsch reaction. In the final step, the synthesis of thieno[2,3‐*c*]pyridine derivatives was achieved in good yield via acid‐mediated denitrogenative transformation reactions. This mild condition synthetic process has enabled access to the title compounds, of which limited examples have been reported until now in the literature. Thus, the syntheses of 7‐(substituted methyl)thieno[2,3‐*c*]pyridine, thieno[2,3‐*c*]pyridine‐7‐ylmethyl esters, and imidazo[1,5‐*ɑ*]thieno[2,3‐*c*]pyridine derivatives have been successfully achieved.

## Graphical abstract

Thieno[2,3‐*c*]pyridine derivatives are remarkable skeletons in pharmaceutical and material science studies in modern organic chemistry. This article reports the synthesis of 7‐(substituted methyl)thieno[2,3‐*c*]pyridine, thieno[2,3‐*c*]pyridine‐7‐ylmethyl ester, and imidazo[1,5‐*ɑ*]thieno[2,3‐*c*]pyridine derivatives through denitrogenative transformation via the intermediate thieno[2,3‐*c*][1,2,3]triazolo[1,5‐*ɑ*]pyridine, using a metal‐free method. In addition to the synthesis of these novel derivatives, an efficient method for the synthesis of this scaffold is also presented.

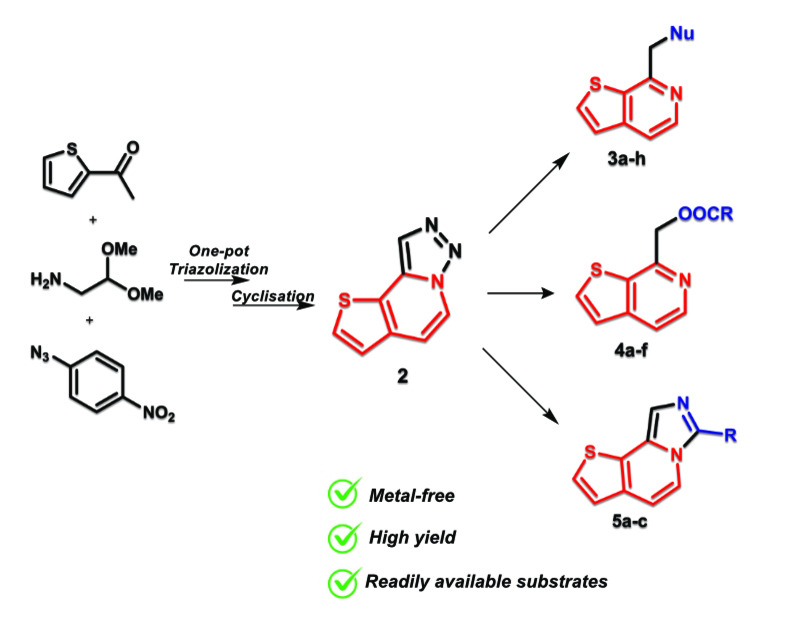

## Introduction

**Figure 1:**
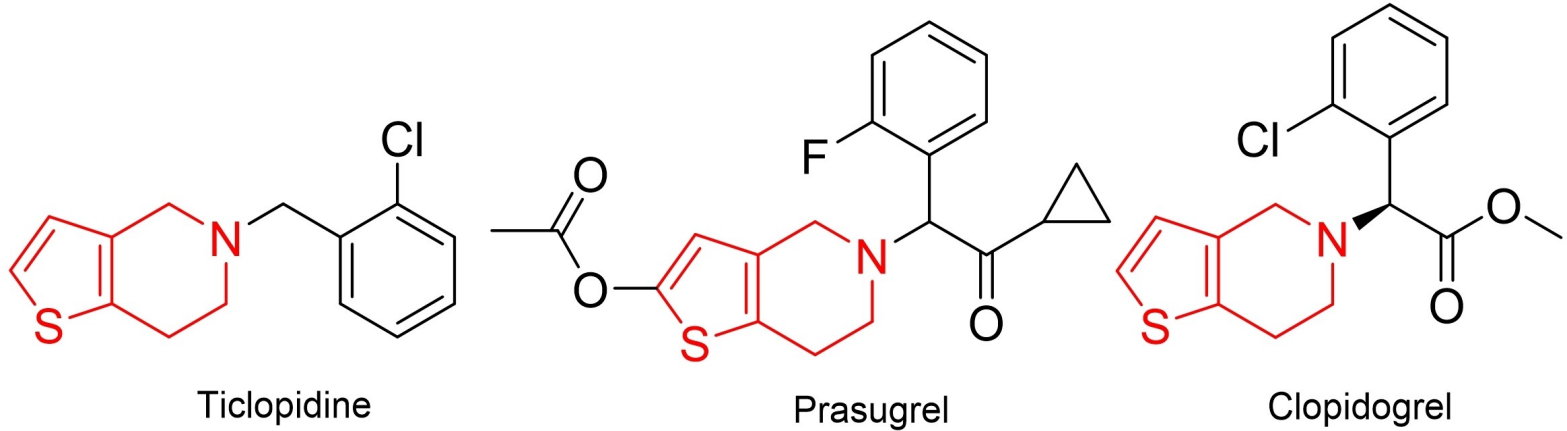
Anticoagulant drugs containing the thienopyridine skeleton.

Thienopyridines are becoming an important class of heterocycles for modern organic chemistry. These compounds, which belong to a larger class of fused pyridine analogues, stand out from other analogues by their diverse biological activities.[^1^, ^2^] In addition to the anticoagulant properties (Figure 1) associated with this skeleton, it has been determined that other analogs have pharmaceutical significance due to their antitumor,[^3^, ^4^] anticancer,[^5^, ^6^] c‐Src inhibitor,^[7]^ antagonist,[^8^, ^9^] and antimicrobial effects.[^10^, ^11^] Among the isomers of thienopyridine, thieno[2,3‐*c*]pyridine has attracted attention in activity studies, due to its presence in the core structure of various kinase inhibitors.[^8^, ^12^] In addition to its biological activity, this isomer has demonstrated a broad application potential, drawing interest in material chemistry due to its electrochemical and photophysical properties.[^13^, ^14^] Therefore, research related to this structure has gained significant importance.

**Scheme 1:**
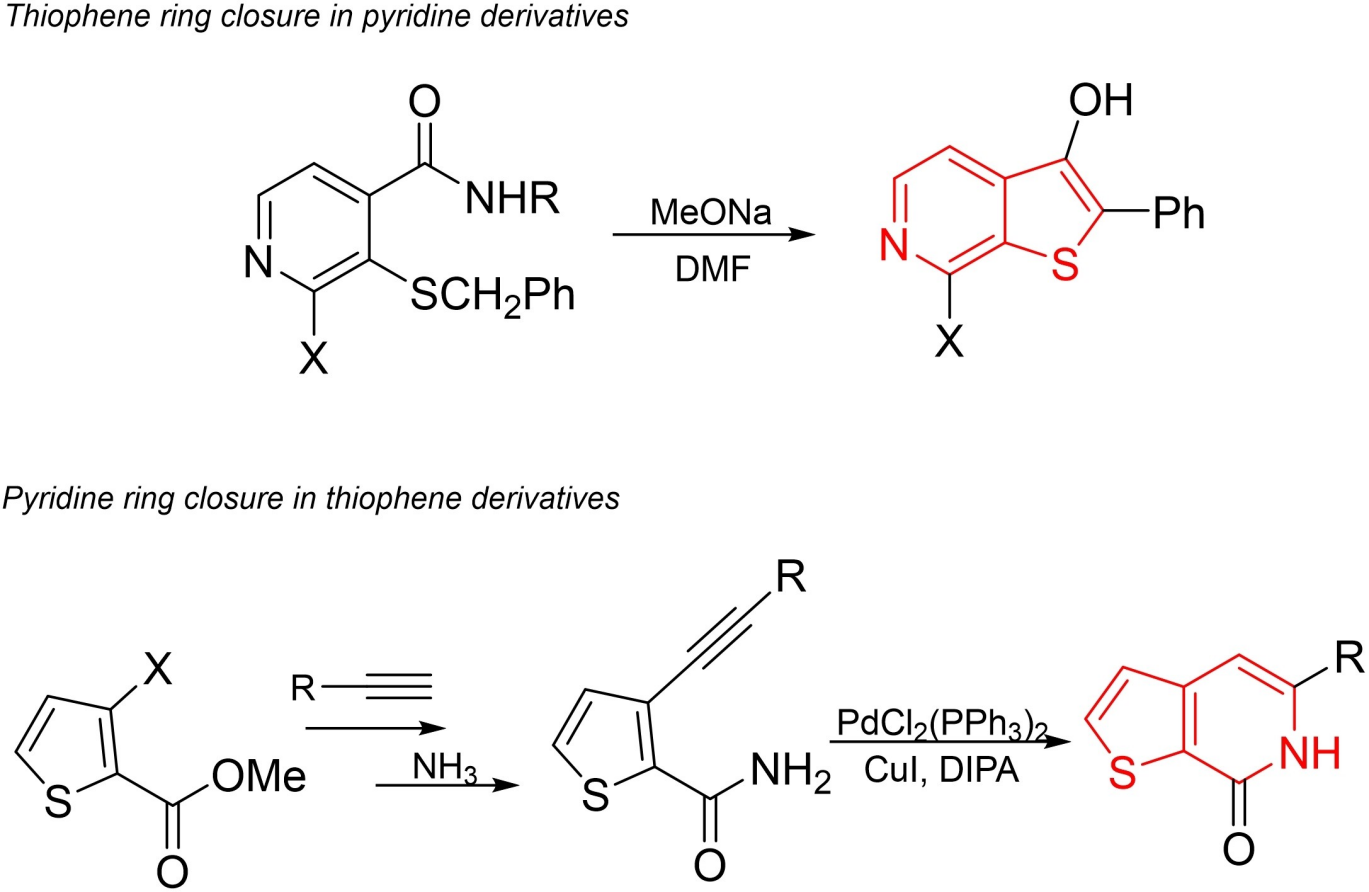
Conventional methods for synthesis of thieno[2,3‐*c*]pyridines.

There are two general strategies used as conventional methods for the synthesis of thieno[2,3‐*c*]pyridine derivatives.^[15]^ One of these strategies involves the closure of the thiophene ring based on pyridine derivatives, while the other involves the closure of the pyridine ring based on thiophene derivatives (Scheme 1).^[16]^ As evident from these examples, the specific starting materials used in these methods limit the diversity of the products, and the use of metal catalysts makes the synthesis expensive and more challenging.

**Scheme 2:**
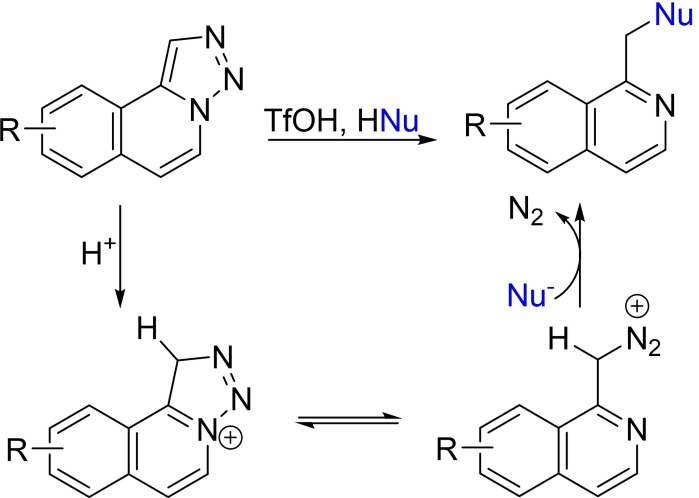
Proposed mechanism of the synthesis of isoquinoline compounds via the acid‐mediated denitrogenative ring‐opening of triazoles.

In 2020, one of us developed an effective method for the synthesis of isoquinoline derivatives by synthesizing 1,2,3‐triazole compounds using a metal‐free three‐component one‐pot triazolation^[17]^ and modified Pomeranz‐Fritsch reaction, and then generating substituted isoquinolines via acid‐mediated denitrogenation of these triazoles in the presence of nucleophiles (Scheme 2).[^18^, ^19^] This approach allowed the synthesis of various isoquinoline compounds, including alkaloid derivatives and analogues with antiviral activities.^[20]^ In our study, we used this method in a straightforward approach towards substituted thieno[2,3‐*c*]pyridine derivatives that are rarely reported in the literature.

## Results and Discussion

**Scheme 3:**
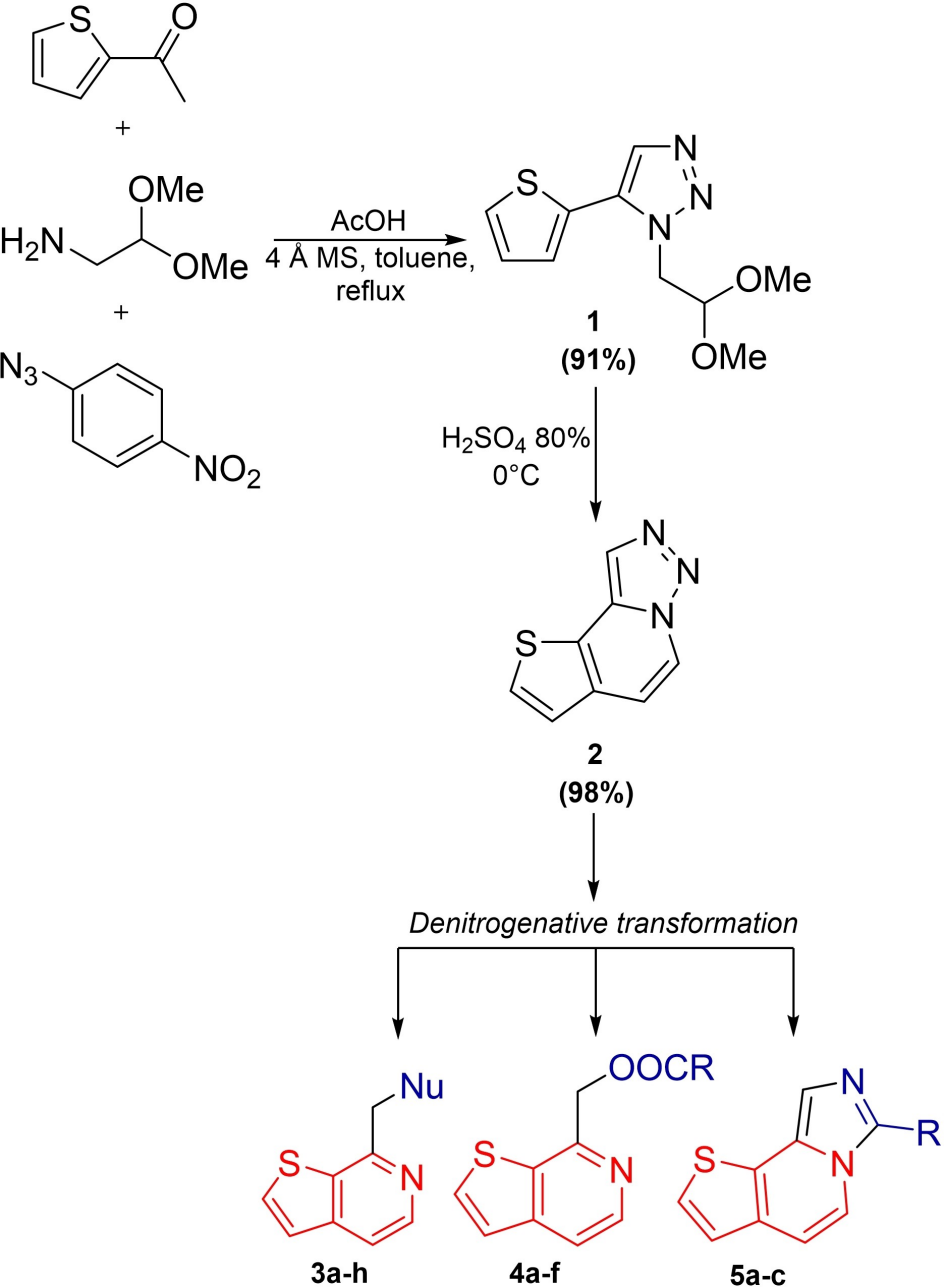
Our approach for the synthesis of thieno[2,3‐*c*]pyridine derivatives.

Thieno[2,3‐*c*]pyridine derivatives were synthesized from the readily available starting material 2‐acetylthiophene through one‐pot triazolization reaction followed by semi‐quantitative Pomeranz‐Fritsch cyclisation reaction of acetal **1**, and finally a denitrogenative transformation of the resulting fused 1,2,3‐triazole compound **2** (Scheme 3).

Through the denitrogenative transformation reaction, the synthesis of 7‐(substituted methyl)thieno[2,3‐*c*]pyridines and thieno[2,3‐*c*]pyridine‐7‐methyl esters via a nucleophilic insertion mechanism, and imidazo[1,5‐*ɑ*]thieno[2,3‐*c*]pyridine derivatives via a transannulation mechanism has been achieved (Scheme 3). This method applied to the synthesis of thienopyridine derivatives has provided numerous advantages: i) we avoid metal catalysts, making the procedure more environmentally friendly and avoiding toxic residues in compounds with potential pharmaceutical activity, ii) the synthesis is cost‐effective, iii) it uses simple and readily available reactants, and iv) it overcomes the limitations of other methods through late‐stage derivatization.

As a starting point for the synthesis of substituted thienopyridines, we chose butanol as a model nucleophile and refluxed it under nitrogen atmosphere for overnight in the presence of PTSA (para‐toluenesulfonic acid) as a catalyst, using different solvents (Table 1). Under these conditions, the highest yield was obtained with 1,2‐DCE (1,2‐dichloroethane) as the solvent (entry 3). In the continuation of optimization studies, reactions were repeated with the same solvents using TfOH (trifluoromethanesulfonic acid) as the catalyst, and higher product yields were observed with this catalyst. The yield of the desired product **3 c** could be improved to an acceptable level using TfOH as the catalyst and DCE as the solvent, lowering the reaction temperature to 80 °C (entry 6).

**Table 1:** Optimization of the reaction conditions.^[a]^

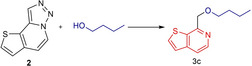					
Entry	Catalyst	Solvent	T [°C]	Time [h]	Yield^[b]^ **3 c** [%]
**1**	PTSA	Toluene	110	24	2
**2**	PTSA	Dioxane	100	24	5
**3**	PTSA	1,2‐DCE	80	24	20
**4**	TfOH	Toluene	110	24	11
**5**	TfOH	Dioxane	100	24	48
**6**	TfOH	1,2‐DCE	80	24	72
**7**	–	1,2‐DCE	80	24	0
**8**	–	1,2‐DCE	80	48	0

[a] Reaction conditions: Compound **2** (1 eq.), butan‐1‐ol (10 eq.), catalyst (2 eq.), under a N_2_ atmosphere. [b] The yields were determined by NMR analysis of the crude product using 1,3,5‐trimethoxybenzene as an internal standard.

Having established the optimal conditions we synthesized obtained 7‐(substituted methyl)thieno[2,3‐c]pyridines derivatives as a result of the reaction of the compound **2** and a nucleophile via the nucleophilic insertion mechanism (Table 2). Here, the desired products (**3 b**–**3 f**) were obtained in good yield through denitrogenative transformation using five different OH nucleophiles including methanol, primary and secondary alcohols and phenols. The useful bromide analog **3 a** was synthesized under the same conditions using tetrabutylammonium bromide (TBAB). Products **3 g** and **3 h** were obtained as a result of the insertion of the used acid catalysts. Since these acids are not very nucleophilic, the isolated yields are in these cases somewhat lower.

**Table 2:**
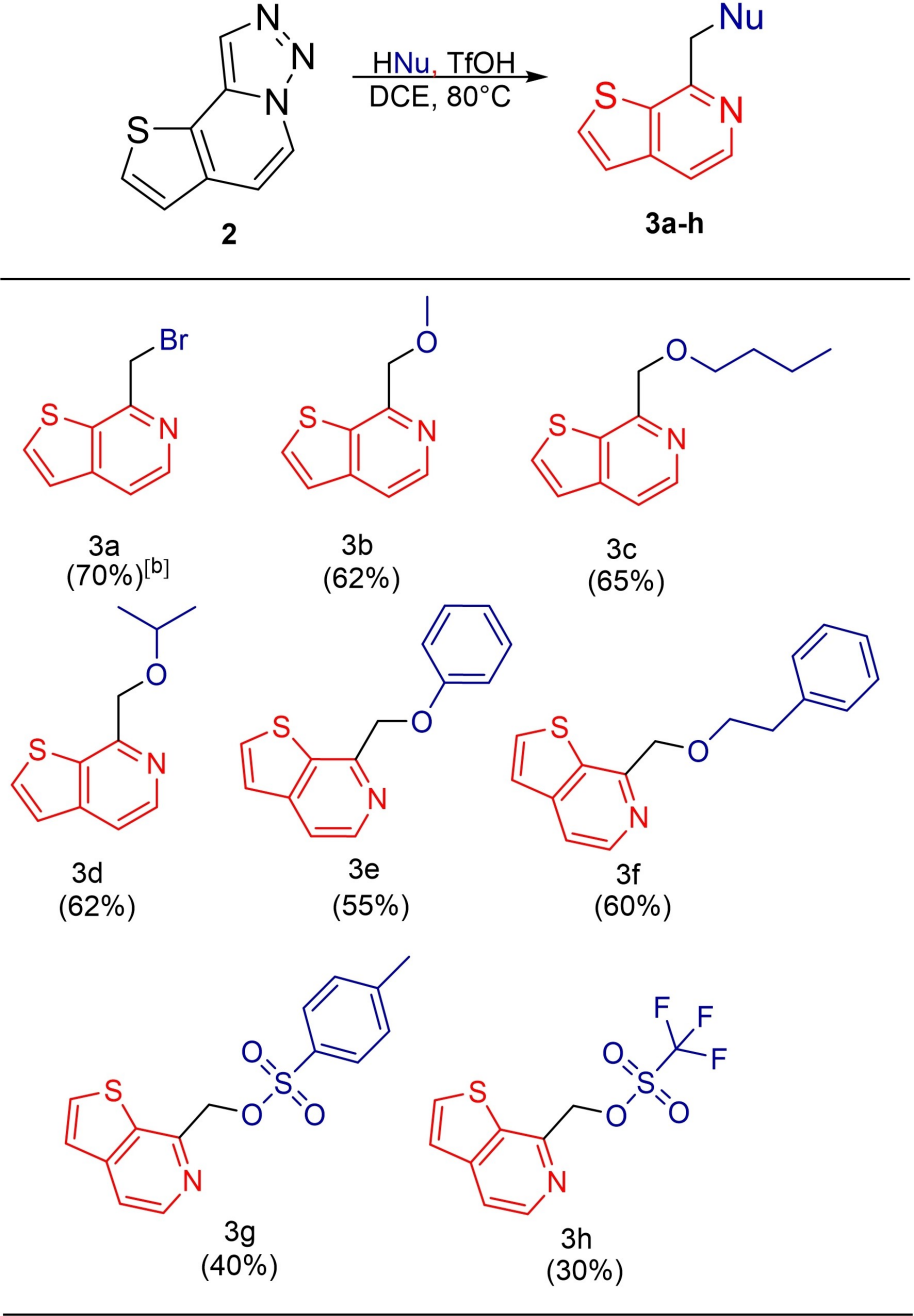
Synthesis of 7‐(substituted methyl)thieno[2,3‐*c*]pyridines.^[a]^

[a] Isolated yields. [b] Reaction conditions for **3 a**: Tetrabutylammonium bromide.

Based on this, when we repeated the reaction with liquid carboxylic acids as the solvent, we observed the formation of ester products. This ester formation reaction was carried out at 100 °C for 1–3 hours with good yield. As a result, we obtained thieno[2,3‐*c*]pyridine‐7‐ylmethyl ester derivatives (**4 b**–**4 f**) with different acids (Table 3). Additionally, the alcohol **4 a** was synthesized by reacting with 1 M H₂SO₄/H₂O.

**Table 3:**
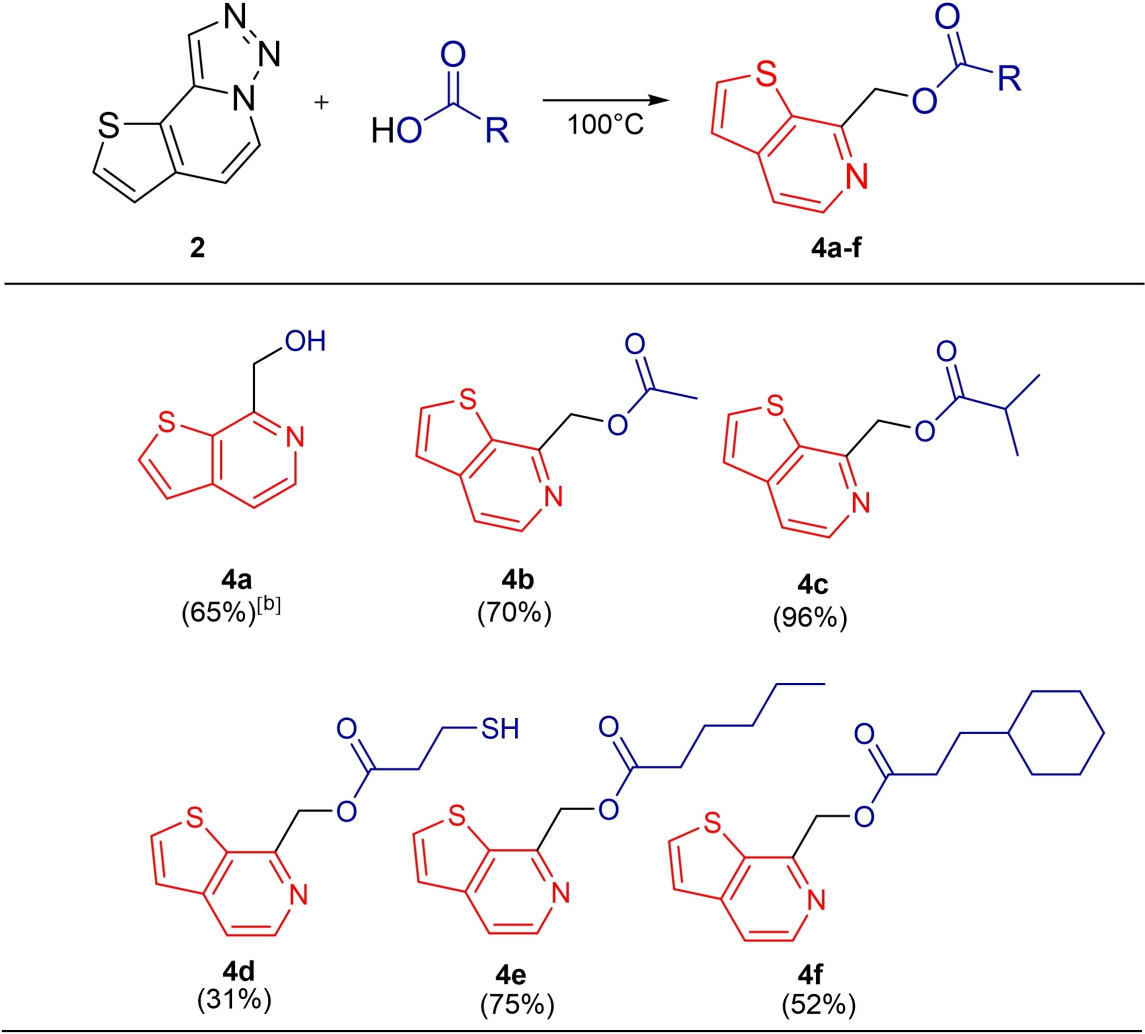
Synthesis of thieno[2,3‐*c*]pyridine‐7‐ylmethyl esters.^[a]^

[a] Reaction conditions: Compound **2** (0.25 mmol), carboxylic acid (2 mL). [b] Reaction conditions for **4 a**: Compound **2** (0.25 mmol), 1 M H_2_SO_4_/H_2_O (2 mL).

In another derivatization study, we used nitrile compounds as the nucleophile under the optimized conditions. As a result of this reaction, imidazo[1,5‐*ɑ*]thieno[2,3‐*c*]pyridine derivatives (**5 a**–**5 c)** derivatives were obtained through a transannulation mechanism probably involving a nitrilium intermediate that recyclizes with the pyridine nitrogen. ^[21]^ (Table 4).

**Table 4:**
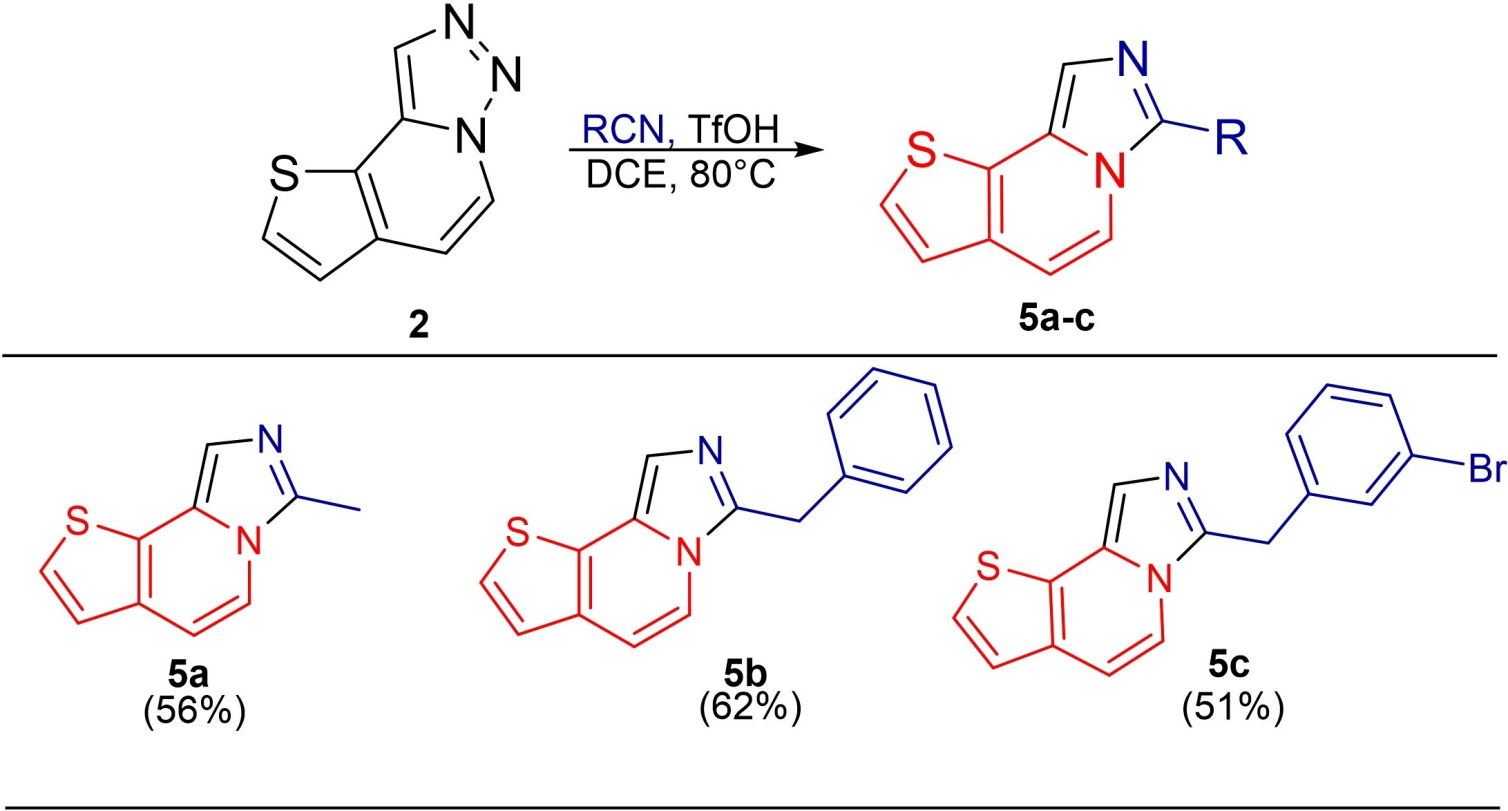
Synthesis of imidazo[1,5‐*ɑ*]thieno[2,3‐*c*]pyridines.

## Conclusions

We have developed a new and effective strategy for the synthesis of thieno[2,3‐*c*]pyridine derivatives, which may be important due to their biological activities, and this will be the subject of follow‐up studies. Using this method, a small library of novel thienopyridine derivatives has been synthesized starting from fused 1,2,3‐triazoles under mild conditions and without metal catalysts, via different mechanisms. Thus, a new method for synthesizing this important class of compounds has been presented, and the limitations of derivatization in conventional methods have also been overcome.

## Conflict of Interests

The authors declare no conflict of interest.

1

## Supporting information

As a service to our authors and readers, this journal provides supporting information supplied by the authors. Such materials are peer reviewed and may be re‐organized for online delivery, but are not copy‐edited or typeset. Technical support issues arising from supporting information (other than missing files) should be addressed to the authors.

Supporting Information

## Data Availability

The data that support the findings of this study are available in the supplementary material of this article.
