# Supplementary material for: Synthesis of Thieno[2,3‐c]pyridine Derivatives by 1,2,3‐Triazole‐Mediated Metal‐Free Denitrogenative Transformation Reaction
Source: ChemistryOpen. 2025 Mar 15;14(5):e202500060. doi: 10.1002/open.202500060 (PMC12075097; doi:10.1002/open.202500060)

ChemistryOpen

Supporting Information

Synthesis of Thieno[2,3-*c*]pyridine Derivatives by 1,2,3-Triazole-Mediated Metal-Free Denitrogenative Transformation Reaction

Kumsal Eroğlu, Ömer Tahir Günkara,* and Wim Dehaen

Supporting Information

Synthesis of Thieno[2,3-*c*]pyridine Derivatives by 1,2,3-Triazole-Mediated Metal-Free Denitrogenative Transformation Reaction

Kumsal Eroğlu,[b] Ömer Tahir Günkara,*[b] and Wim Dehaen[a]

[a]Department of Chemistry, KU Leuven, Celestijnenlaan 200F B-3001 Leuven, Belgium.

[b]Department of Chemistry, Yıldız Technical University, Davutpasa, Esenler, 34220, Istanbul, Türkiye

E-mail: omerrrgunkara@hotmail.com

Contents

Experimental procedures and characterization data S2

General information S2

One-pot triazolization procedure for the synthesis of 1-(2,2-dimethoxyethyl)-5-(thiophen-2-yl)-1*H*-1,2,3-triazole (**1**) S2

Modified Pomeranz-Fritsch procedure for the synthesis of thieno[2,3-*c*][1,2,3]triazolo[1,5-*a*]pyridine (**2**) S2

General procedure for the triflic acid-mediated ring opening of thieno[2,3-*c*][1,2,3]triazolo[1,5-*a*]pyridine toward 7-(substituted methyl)thieno[2,3-*c*]pyridine (**3a-h**) S3

General procedure for synthesis of thieno[2,3-*c*]pyridine-7-ylmethyl ester derivatives (**4a-f**) S5

General procedure for synthesis of imidazo[1,5-*a*]thieno[2,3-*c*]pyridine derivatives (**5a-c**) S7

NMR spectra S9

Experimental procedures and characterization data

General information

All the solvents and reagents were commercially available from Sigma-Aldrich, Acros Organics, Alfa Aesar, and Fisher Scientific, and used as received. For column chromatography, 60-230 mesh silica 60 (Acros) was used as the stationary phase. NMR spectra were recorded on commercial instruments (Bruker 500 MHz) and chemical shifts (δ) were reported in parts per million (ppm) referenced to tetramethylsilane (^{1}H), or the internal (NMR) solvent signal (^{13}C). High-resolution mass spectra were acquired on Agilent G6530B TOF/Q-TOF Mass spectrometer.

One-pot triazolization procedure for the synthesis of 1-(2,2-dimethoxyethyl)-5-(thiophen-2-yl)-1*H*-1,2,3-triazole (1)

In an oven-dried Schlenk reaction tube equipped with a magnetic stirring bar were added the ketone (2.5 mmol, 1 eq.), aminoacetaldehyde dimethyl acetal (5 mmol, 2 eq.) and AcOH (2.5 mmol, 1 eq.) under nitrogen gas and dissolved in toluene (2 ml). 4-Nitrophenylazide (3.75 mmol) was dissolved in toluene (1.5 ml) and added through the walls of the tube. The reaction was stirred at 100°C under reflux overnight. Purified by column chromatography to a yellowish-orange viscous oil (SiO_2, DCM:Ethyl Acetate (4 :1)). Yield: 91%.

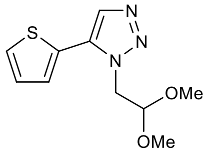

1

^{1}H NMR (400 MHz, $CDCl_3$) δ 7.74 (s, 1H), 7.48 (d, J = 5.2 Hz, 1H), 7.36 (d, J = 4.2 Hz, 1H), 7.15 (dd, J = 5.4, 3.9 Hz, 1H), 4.84 (t, J = 5.7 Hz, 1H), 4.49 (d, J = 5.6 Hz, 2H), 3.35 (s, 6H).
^{13}C NMR (101 MHz, $CDCl_3$) δ 133.17, 132.42, 128.98, 128.04, 127.91, 126.32, 103.16, 55.15, 49.69.
HRMS (Q-TOF): *m/z* $[M + H]^+$ calculated for $C_{10}H_{13}N_3O_2S$: 240.08; found: 240.0814

Modified Pomeranz-Fritsch procedure for the synthesis of thieno[2,3-*c*][1,2,3]triazolo[1,5-*a*]pyridine (2)

Triazole **1** (0.6 mmol) was dissolved in methanol (0.2 ml) and H_2SO_4 (4 ml, 80%) was added while keeping the mixture in an ice bath at 0°C. The reaction mixture was stirred at room temperature for 6 hours. The reaction mixture was then purified with ice. It was neutralized with 3 M NaOH at 0°C. The mixture was extracted with DCM and dried. The pure sand-colored solid was thus obtained; yield: 98%, mp 78-80 °C.

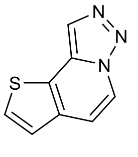

2

^{1}H NMR (400 MHz, $CDCl_3$) δ 8.62 (dd, J = 7.3, 1.0 Hz, 1H), 8.19 (d, J = 1.0 Hz, 1H), 7.64 (d, J = 5.2 Hz, 1H), 7.43 (d, J = 5.2 Hz, 1H), 7.32 (d, J = 7.3 Hz, 1H).
^{13}C NMR (101 MHz, $CDCl_3$) δ 136.17, 130.79, 128.79, 127.45, 124.89, 124.28, 122.50, 111.91.
HRMS (Q-TOF): *m/z* $[M + H]^+$ calculated for $C_8H_5N_3S$: 176.02; found: 176.0274

General procedure for the triflic acid-mediated ring opening of thieno[2,3-*c*][1,2,3]triazolo[1,5-*a*]pyridine toward 7-(substituted methyl)thieno[2,3-*c*]pyridine (3a-h)

To a dried Schlenk reaction tube under nitrogen gas, thieno[2,3-*c*][1,2,3]triazolo[1,5-*a*]pyridine (**2**) and a nucleophile (10 eq.) were added and dissolved in DCE (7.5 mL). After 5 minutes, trifluoromethanesulfonic acid (triflic acid, TfOH) (1.5 eq.) was added, and the reaction was stirred at 100°C for 1 day. When the mixture reaches room temperature, a saturated solution of $NaHCO_3$ (10 mL) was added. The mixture was extracted with DCM (20 mL, 3x) and dried. The thienopyridine was then purified by column chromatography and/or p-TLC.

7-(Bromomethyl)thieno[2,3-*c*]pyridine (3a)

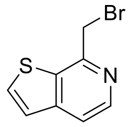

Prepared according to the general procedure for the triflic acid-mediated ring opening, using compound **2** (0.46 mmol) and TBAB (4.6 mmol). Column chromatography was carried out using a DCM/EtOAc mixture (4:1) as the eluent. This afforded a brown-red viscous oil; yield: 70%.

^{13}C NMR (126 MHz, $CDCl_3$) δ 150.76, 146.27, 142.93, 134.92, 131.96, 123.51, 118.05, 46.20.
^{1}H NMR (500 MHz, $CDCl_3$) δ 8.49 (d, *J* = 5.5 Hz, 1H), 7.75 (d, *J* = 5.3 Hz, 1H), 7.69 (d, *J* = 5.3 Hz, 1H), 7.43 (d, *J* = 5.3 Hz, 1H), 4.97 (s, 2H).
HRMS (Q-TOF): *m/z* $[M + H]^+$ calculated for C_8H_6BrNS : 227.11, found 227.1

7-(Methoxymethyl)thieno[2,3-*c*]pyridine (3b)

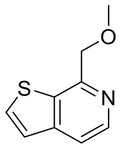

Prepared according to the general procedure for the triflic acid-mediated ring opening, using compound **2** (0.46 mmol) and methanol (4.6 mmol). Column chromatography was carried out using a DCM/EtOAc mixture (4:1) as the eluent. This afforded a yellow viscous oil; yield: 62%.

^{1}H NMR (500 MHz, $CDCl_3$) δ 8.44 (d, *J* = 5.5 Hz, 1H), 7.71 (d, *J* = 5.3 Hz, 1H), 7.64 (d, *J* = 5.5 Hz, 1H), 7.38 (d, *J* = 5.3 Hz, 1H), 4.91 (s, 2H), 3.49 (s, 3H).
^{13}C NMR (126 MHz, $CDCl_3$) δ 153.06, 146.03, 142.38, 133.54, 132.89, 122.73, 117.16, 75.78, 58.93.
HRMS (Q-TOF): *m/z* $[M + H]^+$ calculated for C_9H_9NOS : 180.04, found 180.0473.

7-(Butoxymethyl)thieno[2,3-*c*]pyridine (3c)

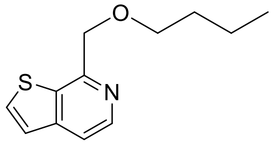

Prepared according to the general procedure for the triflic acid-mediated ring opening, using compound **2** (0.46 mmol) and 1-butanol (4.6 mmol). Column chromatography was carried out using a DCM/EtOAc mixture (4:1) as the eluent. This afforded a yellow viscous oil; yield: 65%.

^{1}H NMR (500 MHz, $CDCl_3$) δ 8.42 (d, *J* = 5.3 Hz, 1H), 7.71 (d, *J* = 5.3 Hz, 1H), 7.62 (d, *J* = 5.3 Hz, 1H), 7.36 (d, *J* = 5.3 Hz, 1H), 4.94 (s, 2H), 3.58 (t, *J* = 6.6 Hz, 2H), 1.73 – 1.66 (m, 2H), 1.44 (dq, *J* = 14.8, 7.4 Hz, 2H), 0.93 (t, *J* = 7.4 Hz, 3H).
^{13}C NMR (126 MHz, $CDCl_3$) δ 153.59, 146.06, 142.21, 133.57, 133.07, 74.32, 71.30, 31.77, 19.36, 13.95.

HRMS (Q-TOF): *m/z* [M + H]+ calculated for $C_{12}H_{15}NOS$: 222.09, found 222.0941.

7-(Isopropoxymethyl)thieno[2,3-*c*]pyridine (3d)

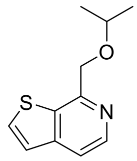

Prepared according to the general procedure for the triflic acid-mediated ring opening, using compound **2** (0.46 mmol) and 2-propanol (4.6 mmol). Column chromatography was carried out using a DCM/EtOAc mixture (4:1) as the eluent. This afforded a yellow viscous oil; yield: 62%.

^{1}H NMR (500 MHz, $CDCl_3$) δ 8.41 (d, *J* = 5.5 Hz, 1H), 7.71 (d, *J* = 5.5 Hz, 1H), 7.62 (d, *J* = 5.3 Hz, 1H), 7.36 (d, *J* = 5.5 Hz, 1H), 4.95 (s, 2H), 3.81 (h, *J* = 6.1 Hz, 1H), 1.31 (d, *J* = 6.1 Hz, 6H).
^{13}C NMR (126 MHz, $CDCl_3$) δ 153.81, 146.07, 142.15, 133.08, 122.53, 116.98, 72.30, 71.57, 21.94.
HRMS (Q-TOF): *m/z* [M + Na]+ calculated for $C_{11}H_{13}NOS$: 230.06, found 230.0602.

7-(Phenoxymethyl)thieno[2,3-*c*]pyridine (3e)

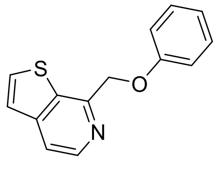

Prepared according to the general procedure for the triflic acid-mediated ring opening, using compound **2** (0.46 mmol) and phenol (4.6 mmol). Column chromatography was carried out using a DCM/EtOAc mixture (4:1) as the eluent. This afforded a yellow viscous oil; yield: 55%.

^{1}H NMR (500 MHz, $CDCl_3$) δ 8.48 (d, *J* = 5.5 Hz, 1H), 7.72 (d, *J* = 5.5 Hz, 1H), 7.67 (d, *J* = 5.5 Hz, 1H), 7.38 (d, *J* = 5.5 Hz, 1H), 7.31 – 7.27 (m, 2H), 7.08 (dd, *J* = 8.9, 1.1 Hz, 2H), 6.97 (t, *J* = 7.3 Hz, 1H), 5.52 (s, 2H).
^{13}C NMR (126 MHz, $CDCl_3$) δ 158.19, 151.95, 146.30, 142.32, 133.18, 129.55, 122.67, 121.42, 117.48, 114.79, 71.18.
HRMS (Q-TOF): *m/z* [M + H]+ calculated for $C_{14}H_{11}NOS$: 242.06, found 242.0632.

7-(Phenethoxymethyl)thieno[2,3-*c*]pyridine (3f)

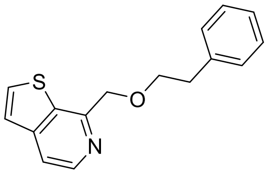

Prepared according to the general procedure for the triflic acid-mediated ring opening, using compound **2** (0.46 mmol) and 2-phenylethan-1-ol (4.6 mmol). Column chromatography was carried out using a DCM/EtOAc mixture (4:1) as the eluent. This afforded a yellow viscous oil; yield: 60%.

^{1}H NMR (500 MHz, $CDCl_3$) δ 8.42 (d, *J* = 5.3 Hz, 1H), 7.70 (d, *J* = 5.3 Hz, 1H), 7.63 (d, *J* = 5.5 Hz, 1H), 7.37 (d, *J* = 5.5 Hz, 1H), 7.29 – 7.18 (m, 5H), 4.97 (s, 2H), 3.80 (t, *J* = 7.3 Hz, 2H), 3.03 (t, *J* = 7.3 Hz, 2H).
^{13}C NMR (126 MHz, $CDCl_3$) δ 153.30, 146.07, 142.26, 138.64, 133.57, 133.05, 129.00, 128.39, 126.29, 122.61, 117.11, 74.33, 72.26, 36.24.
HRMS (Q-TOF): *m/z* [M + H]+ calculated for $C_{16}H_{15}NOS$: 270.09, found 270.0942.

Thieno[2,3-*c*]pyridin-7-ylmethyl 4-methylbenzenesulfonate (3g)

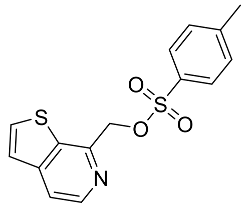

Prepared according to the general procedure for the triflic acid-mediated ring opening, using compound **2** (0.46 mmol) and TfOH (0.69 mmol). Column chromatography was carried out using a DCM/EtOAc mixture (4:1) as the eluent. This afforded a yellow viscous oil; yield: 40%.

^{1}H NMR (500 MHz, $CDCl_3$) δ 8.40 (d, *J* = 5.5 Hz, 1H), 7.84 (d, *J* = 8.4 Hz, 2H), 7.73 (d, *J* = 5.3 Hz, 1H), 7.65 (d, *J* = 5.3 Hz, 1H), 7.38 (d, *J* = 5.5 Hz, 1H), 7.29 (d, *J* = 7.8 Hz, 2H), 5.44 (s, 2H), 2.42 (s, 3H).
^{13}C NMR (126 MHz, $CDCl_3$) δ 147.84, 146.33, 145.08, 142.36, 134.76, 132.81, 132.45, 129.78, 128.21, 123.07, 118.22, 71.92, 21.66.
HRMS (Q-TOF): *m/z* [M + H]$^+$ calculated for $C_{15}H_{13}NO_3S_2$: 320.04, found 320.0404.

Thieno[2,3-*c*]pyridin-7-ylmethyl trifluoromethanesulfonate (3h)

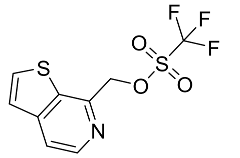

Prepared according to the general procedure for the triflic acid-mediated ring opening, using compound **2** (0.46 mmol) and PTSA (0.69 mmol). Column chromatography was carried out using a DCM/EtOAc mixture (4:1) as the eluent. This afforded a brown viscous oil; yield: 30%.

^{1}H NMR (500 MHz, $CDCl_3$) δ 8.44 (d, *J* = 5.5 Hz, 1H), 7.72 (d, *J* = 5.3 Hz, 1H), 7.64 (d, *J* = 5.6 Hz, 1H), 7.42 (d, *J* = 5.3 Hz, 1H), 5.02 (s, 2H).
^{13}C NMR (126 MHz, $CDCl_3$) δ 153.60, 145.70, 141.68, 132.39, 131.88, 123.37, 117.08, 63.20.
HRMS (Q-TOF): *m/z* [M + H]$^+$ calculated for $C_9H_6F_3NO_3S_2$: 297.98, found 297.9878.

General procedure for synthesis of thieno[2,3-*c*]pyridine-7-ylmethyl ester derivatives (4a-f)

To a dried Schlenk reaction tube under nitrogen gas, thieno[2,3-*c*][1,2,3]triazolo[1,5-*a*]pyridine (**2**) (0.25 mmol) and a carboxylic acid (2 mL) were added, and the reaction was stirred at 100°C for 1-3 hours. When the mixture reaches room temperature, a saturated solution of $NaHCO_3$ (20 mL) was added. The mixture was extracted with DCM (20 mL, 3x) and dried. It was then purified by column chromatography was carried out using a DCM/EtOAc mixture (4:1) as the eluent.

Thieno[2,3-*c*]pyridin-7-ylmethanol (4a)

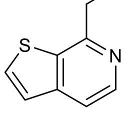

To a dried Schlenk reaction tube under nitrogen gas, thieno[2,3-*c*][1,2,3]triazolo[1,5-*a*]pyridine (**2**) and 1 M H_2SO_4/H_2O (2 mL) were added, and the reaction was stirred at 100°C for 2 hours. When the mixture reaches room temperature, a saturated solution of $NaHCO_3$ (20 mL) was added. The mixture was extracted with DCM (20 mL, 3x) and dried. It was then purified by column chromatography was carried out using a DCM/EtOAc mixture (4:1) as the eluent. This afforded a yellow viscous oil; yield: 65%.

^{1}H NMR (500 MHz, $CDCl_3$) δ 8.44 (d, *J* = 5.5 Hz, 1H), 7.72 (d, *J* = 5.4 Hz, 1H), 7.64 (d, *J* = 5.5 Hz, 1H), 7.41 (d, *J* = 5.4 Hz, 1H), 5.30 (s, 1H), 5.02 (s, 2H).
^{13}C NMR (126 MHz, $CDCl_3$) δ 153.70, 145.71, 141.68, 132.43, 131.92, 123.34, 117.06, 63.29.
HRMS (Q-TOF): *m/z* $[M + H]^+$ calculated for C_8H_7NOS: 166.03, found 166.0314.

Thieno[2,3-*c*]pyridin-7-ylmethyl acetate (4b)

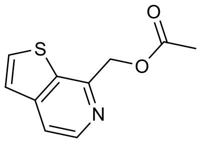

Prepared according to the general procedure, using compound **2** (0.25 mmol) and acetic acid (2 mL). Column chromatography was carried out using a DCM/EtOAc mixture (4:1) as the eluent. This afforded a colorless viscous oil; yield: 70%.

^{1}H NMR (500 MHz, $CDCl_3$) δ 8.49 (d, *J* = 5.4 Hz, 1H), 7.72 (d, *J* = 5.4 Hz, 1H), 7.67 (d, *J* = 5.4 Hz, 1H), 7.41 (d, *J* = 5.4 Hz, 1H), 5.52 (s, 2H), 2.22 (s, 3H).
^{13}C NMR (126 MHz, $CDCl_3$) δ 170.50, 150.33, 146.09, 142.73, 133.92, 132.11, 123.21, 117.56, 66.64, 20.78.
HRMS (Q-TOF): *m/z* $[M + H]^+$ calculated for $C_{10}H_9NO_2S$: 208.04, found 208.0418.

Thieno[2,3-*c*]pyridin-7-ylmethyl isobutyrate (4c)

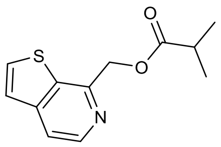

Prepared according to the general procedure, using compound **2** (0.25 mmol) and isobutyric acid (2 mL). Column chromatography was carried out using a DCM/EtOAc mixture (4:1) as the eluent. This afforded a yellow viscous oil; yield: 96%.

^{1}H NMR (500 MHz, $CDCl_3$) δ 8.48 (d, *J* = 5.4 Hz, 1H), 7.72 (d, *J* = 5.4 Hz, 1H), 7.67 (d, *J* = 5.4 Hz, 1H), 7.41 (d, *J* = 5.4 Hz, 1H), 5.51 (s, 2H), 2.74 (hept, *J* = 7.0 Hz, 1H), 1.25 (d, *J* = 7.0 Hz, 6H).
^{13}C NMR (126 MHz, $CDCl_3$) δ 176.64, 150.54, 146.07, 142.64, 134.11, 132.15, 123.15, 117.58, 66.87, 33.98, 19.02.
HRMS (Q-TOF): *m/z* $[M + H]^+$ calculated for $C_{12}H_{13}NO_2S$: 236.07, found 236.0739.

Thieno[2,3-*c*]pyridin-7-ylmethyl 3-mercaptopropanoate (4d)

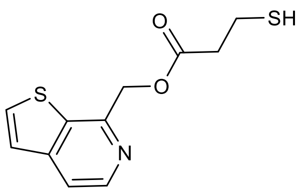

Prepared according to the general procedure, using compound **2** (0.25 mmol) and 3-mercaptopropanoic acid (2 mL). Column chromatography was carried out using a DCM/EtOAc mixture (4:1) as the eluent. This afforded a yellow viscous oil; yield: 31%.

^{1}H NMR (500 MHz, $CDCl_3$) δ 8.49 (d, *J* = 5.5 Hz, 1H), 7.73 (d, *J* = 5.5 Hz, 1H), 7.68 (d, *J* = 5.5 Hz, 1H), 7.42 (d, *J* = 5.3 Hz, 1H), 5.55 (s, 2H), 2.86 – 2.81 (m, 4H), 1.76 – 1.72 (m, 1H).

^{13}C NMR (126 MHz, $CDCl_3$) δ 171.29, 150.17, 146.28, 142.85, 132.27, 123.43, 117.84, 66.87, 38.52, 19.84.
HRMS (Q-TOF): *m/z* $[M + H]^+$ calculated for $C_{11}H_{11}NO_2S_2$: 254.03, found 254.0297.

Thieno[2,3-*c*]pyridin-7-ylmethyl hexanoate (4e)

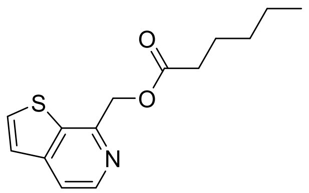

Prepared according to the general procedure, using compound **2** (0.25 mmol) and hexanoic acid (2 mL). Column chromatography was carried out using a DCM/EtOAc mixture (4:1) as the eluent. This afforded a yellow viscous oil; yield: 75%.

^{1}H NMR (500 MHz, $CDCl_3$) δ 8.49 (d, *J* = 5.5 Hz, 1H), 7.72 (d, *J* = 5.3 Hz, 1H), 7.67 (d, *J* = 5.3 Hz, 1H), 7.41 (d, *J* = 5.3 Hz, 1H), 5.52 (s, 2H), 2.48 (t, *J* = 7.6 Hz, 2H), 1.70 (t, *J* = 7.6 Hz, 2H), 1.36 – 1.31 (m, 4H), 0.91 – 0.86 (m, 3H).
^{13}C NMR (126 MHz, $CDCl_3$) δ 172.97, 150.41, 145.99, 142.56, 133.89, 132.06, 123.07, 117.45, 66.44, 33.93, 31.19, 24.43, 22.20, 13.79.
HRMS (Q-TOF): *m/z* $[M + H]^+$ calculated for $C_{14}H_{17}NO_2S$: 264.10, found 264.1047.

Thieno[2,3-*c*]pyridin-7-ylmethyl 3-cyclohexylpropanoate (4f)

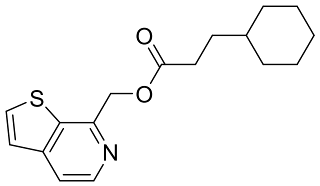

Prepared according to the general procedure, using compound **2** (0.25 mmol) and 3-cyclohexylpropanoic acid (2 mL). Column chromatography was carried out using a DCM/EtOAc mixture (4:1) as the eluent. This afforded a yellow viscous oil; yield: 52%.

^{1}H NMR (500 MHz, $CDCl_3$) δ 8.49 (d, *J* = 5.5 Hz, 1H), 7.72 (d, *J* = 5.3 Hz, 1H), 7.67 (d, *J* = 5.5 Hz, 1H), 7.41 (d, *J* = 5.3 Hz, 1H), 5.51 (s, 2H), 2.52 – 2.46 (m, 2H), 1.70 (t, *J* = 12.6 Hz, 4H), 1.62 – 1.57 (m, 2H), 1.33 – 1.06 (m, 5H), 0.95 – 0.85 (m, 2H).
^{13}C NMR (126 MHz, $CDCl_3$) δ 173.66, 150.54, 146.09, 142.72, 134.00, 132.15, 123.19, 117.56, 66.62, 37.17, 32.96, 32.20, 31.66, 26.53, 26.22.
HRMS (Q-TOF): *m/z* $[M + H]^+$ calculated for $C_{17}H_{21}NO_2S$: 304.13, found 304.1350.

General procedure for synthesis of imidazo[1,5-*a*]thieno[2,3-*c*]pyridine derivatives (5a-c)

To a dried Schlenk reaction tube under nitrogen gas, thieno[2,3-*c*][1,2,3]triazolo[1,5-*a*]pyridine (**2**) and a nitrile (10 eq.) were added and dissolved in DCE (7.5 mL). After 5 minutes, trifluoromethanesulfonic acid (triflic acid, TfOH) (1.5 eq.) was added, and the reaction was stirred at 100°C for 1-3 days. When the mixture reaches room temperature, a saturated solution of $NaHCO_3$ (10 mL) was added. The mixture was extracted with DCM (20 mL, 3x) and dried. It was then purified by column chromatography and/or p-TLC.

7-Methylimidazo[1,5-*a*]thieno[2,3-*c*]pyridine (5a)

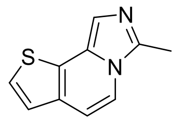

Prepared according to the general procedure for the triflic acid-mediated ring opening, using compound **2** (0.46 mmol) and acetonitrile (4.6 mmol). Preperative thin layer chromatography was carried out using EtOAc/Hexane mixture (5:1) as the eluent. This afforded a yellow viscous oil; yield: 56%.

^{1}H NMR (500 MHz, $CDCl_3$) δ 7.56 (d, *J* = 7.3 Hz, 1H), 7.45 (s, 1H), 7.31 (d, *J* = 5.2 Hz, 1H), 7.21 (d, *J* = 5.2 Hz, 1H), 6.90 (d, *J* = 7.5 Hz, 1H), 2.66 (s, 3H).
^{13}C NMR (126 MHz, $CDCl_3$) δ 136.45, 130.63, 128.64, 126.52, 124.29, 123.87, 118.45, 116.28, 108.85, 12.96.
HRMS (Q-TOF): *m/z* $[M + H]^+$ calculated for $C_{10}H_8N_2S$: 189.04, found 189.0482.

7-Benzylimidazo[1,5-*a*]thieno[2,3-*c*]pyridine (5b)

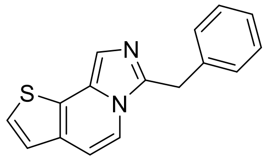

Prepared according to the general procedure for the triflic acid-mediated ring opening, using compound **2** (0.46 mmol) and 2-phenylacetonitrile (4.6 mmol). Preperative thin layer chromatography was carried out using DCM/EtOAc mixture (4:1) as the eluent. This afforded a yellow viscous oil; yield: 62%.

^{1}H NMR (500 MHz, $CDCl_3$) δ 7.55 (s, 1H), 7.51 (d, *J* = 7.5 Hz, 1H), 7.33 (d, *J* = 5.0 Hz, 1H), 7.29 (t, *J* = 7.2 Hz, 2H), 7.23 (d, *J* = 7.3 Hz, 1H), 7.20 (d, *J* = 5.2 Hz, 3H), 6.82 (d, *J* = 7.3 Hz, 1H), 4.46 (s, 2H).
^{13}C NMR (126 MHz, $CDCl_3$) δ 138.44, 136.40, 130.93, 128.85, 128.67, 128.28, 127.03, 126.89, 124.29, 124.14, 118.61, 116.72, 109.03, 33.83.
HRMS (Q-TOF): *m/z* $[M + H]^+$ calculated for $C_{16}H_{12}N_2S$: 265.08, found 265.0790.

7-(3-Bromobenzyl)imidazo[1,5-*a*]thieno[2,3-*c*]pyridine (5c)

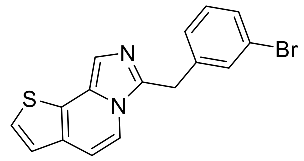

Prepared according to the general procedure, using compound **2** (0.46 mmol) and 2-bromophenylacetonitrile (4.6 mmol). Column chromatography was carried out using a DCM/EtOAc mixture (4:1) as the eluent. This afforded a brown viscous oil; yield: 51%.

^{1}H NMR (500 MHz, $CDCl_3$) δ 7.56 (s, 1H), 7.50 (d, *J* = 7.5 Hz, 1H), 7.37 (d, *J* = 6.7 Hz, 1H), 7.35 (d, *J* = 5.2 Hz, 1H), 7.22 (d, *J* = 5.2 Hz, 1H), 7.15 (t, *J* = 7.7 Hz, 1H), 7.11 (d, *J* = 9.3 Hz, 1H), 6.87 (d, *J* = 7.5 Hz, 1H), 4.42 (s, 2H).
^{13}C NMR (126 MHz, $CDCl_3$) δ 138.74, 137.49, 131.32, 131.02, 130.38, 130.15, 128.65, 127.15, 126.93, 124.31, 122.94, 118.33, 116.92, 109.33, 33.35.
HRMS: *m/z* $[M + H]^+$ calculated for $C_{16}H_{11}BrN_2S$: 342.98, found 342.9890.

NMR spectra

1, ^{1}H, 400 MHz, $CDCl_3$

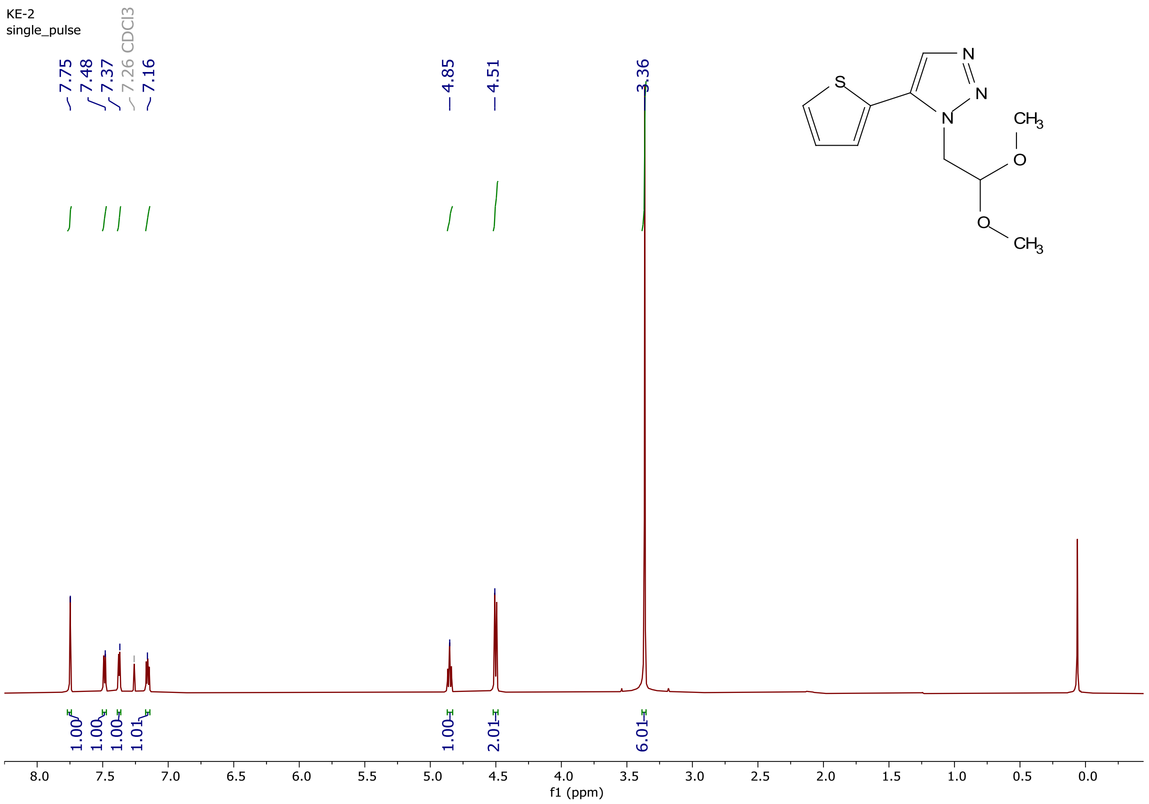

1, ^{13}C, 101 MHz, $CDCl_3$

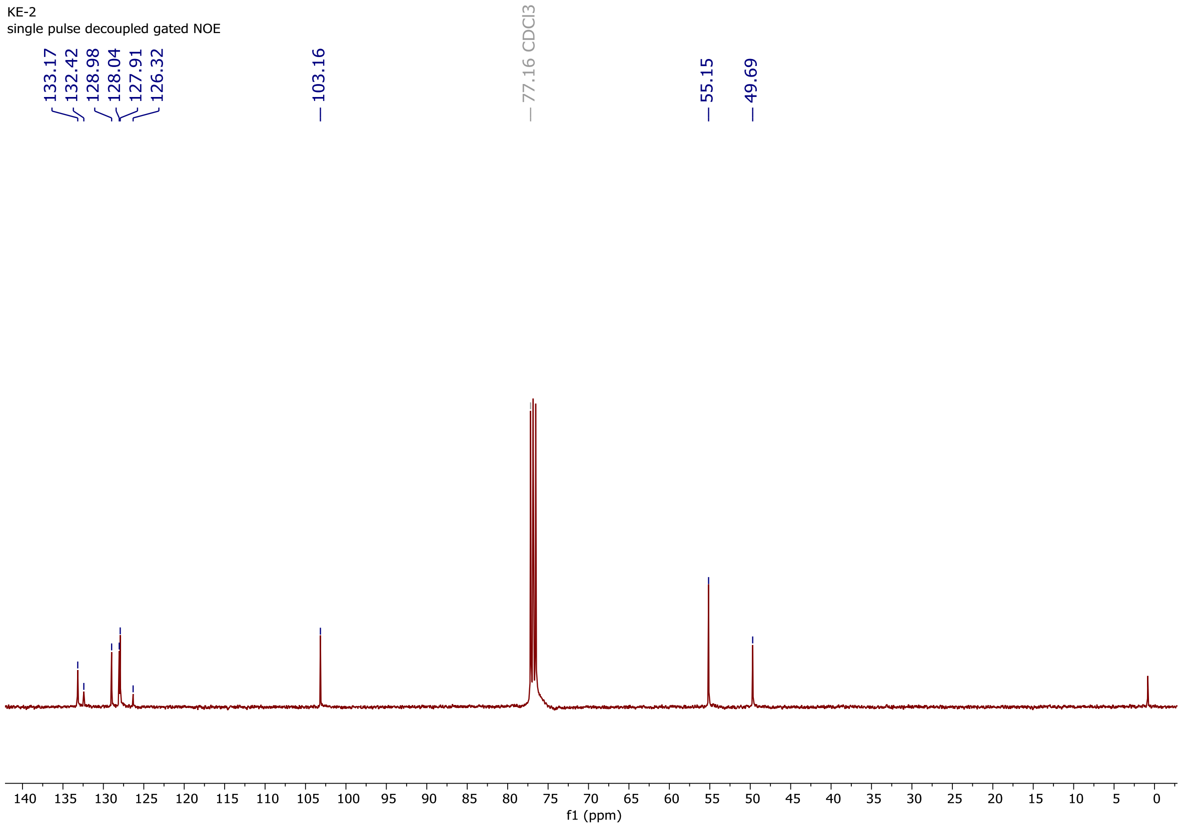

2, ^{1}H, 400 MHz, $CDCl_3$

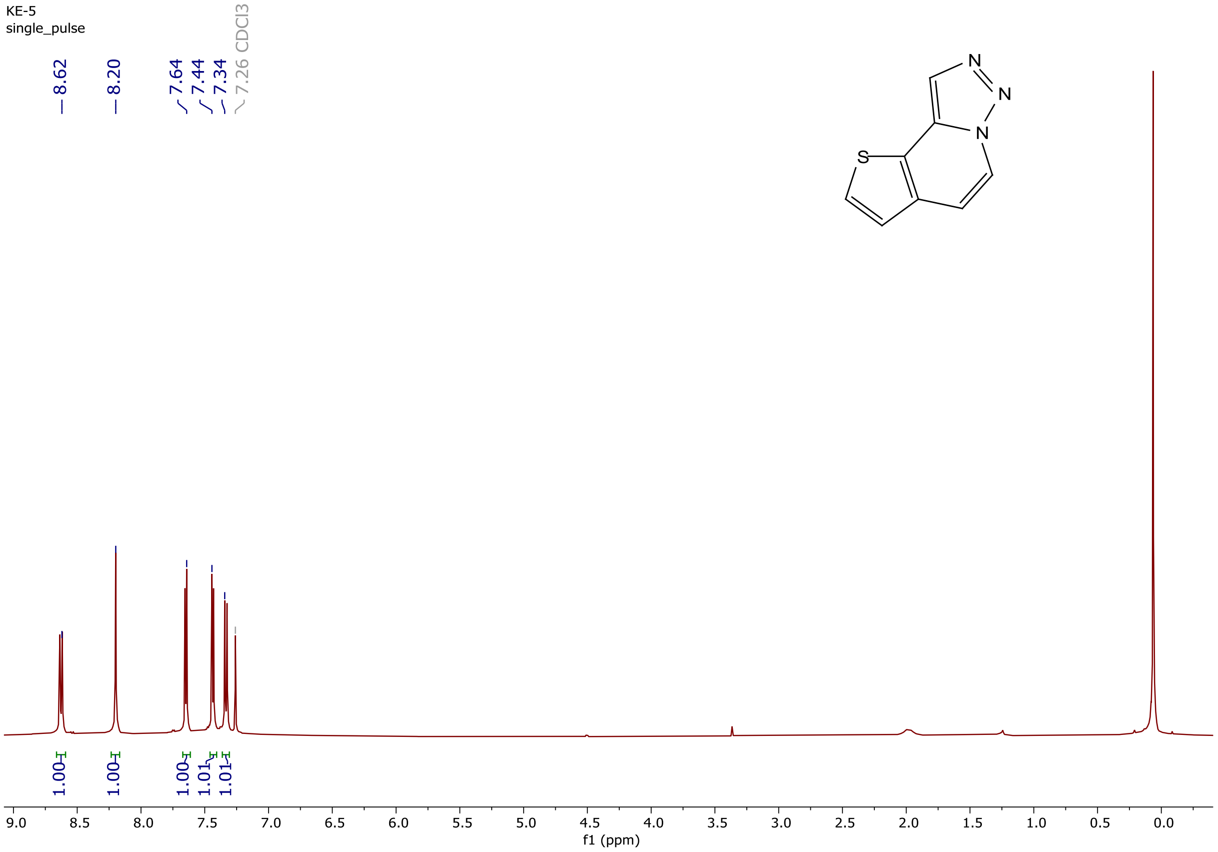

2, ^{13}C, 101 MHz, $CDCl_3$

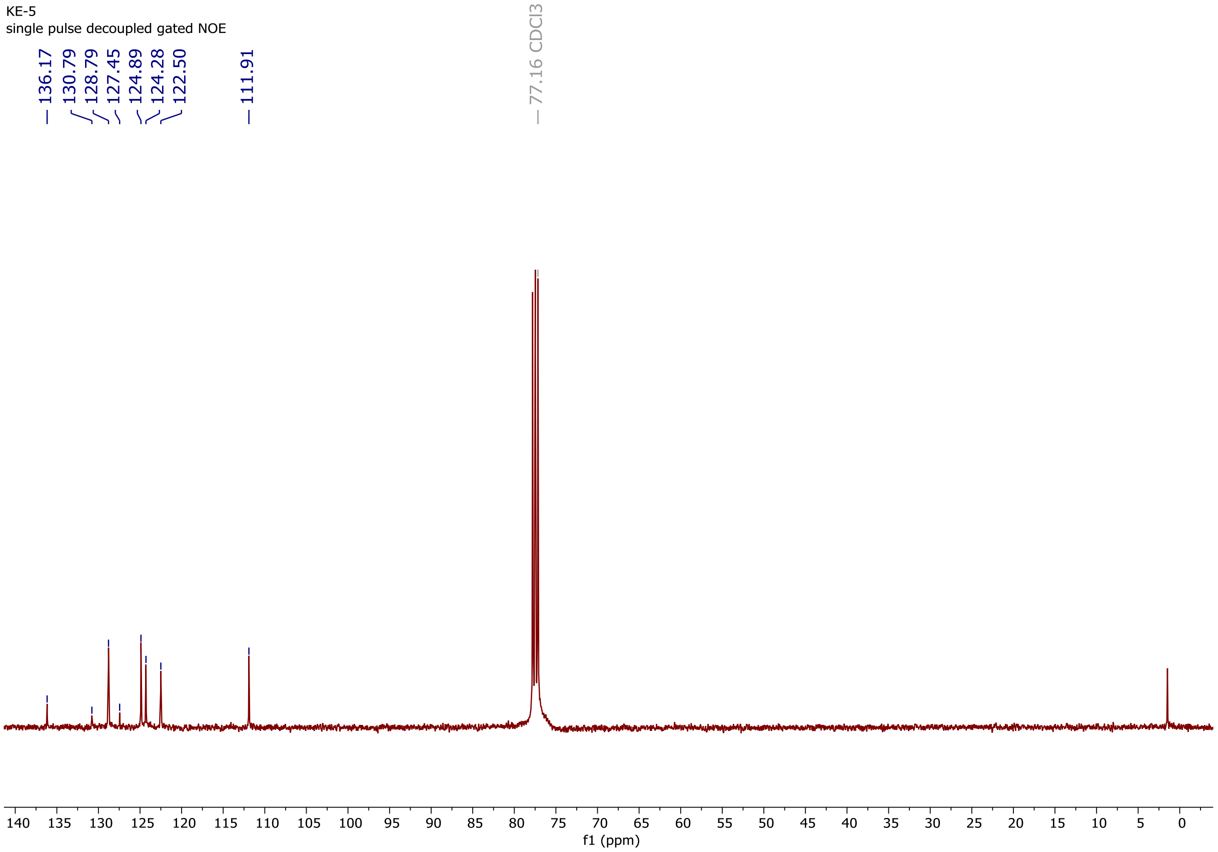

3a, ^{1}H, 500 MHz, $CDCl_3$

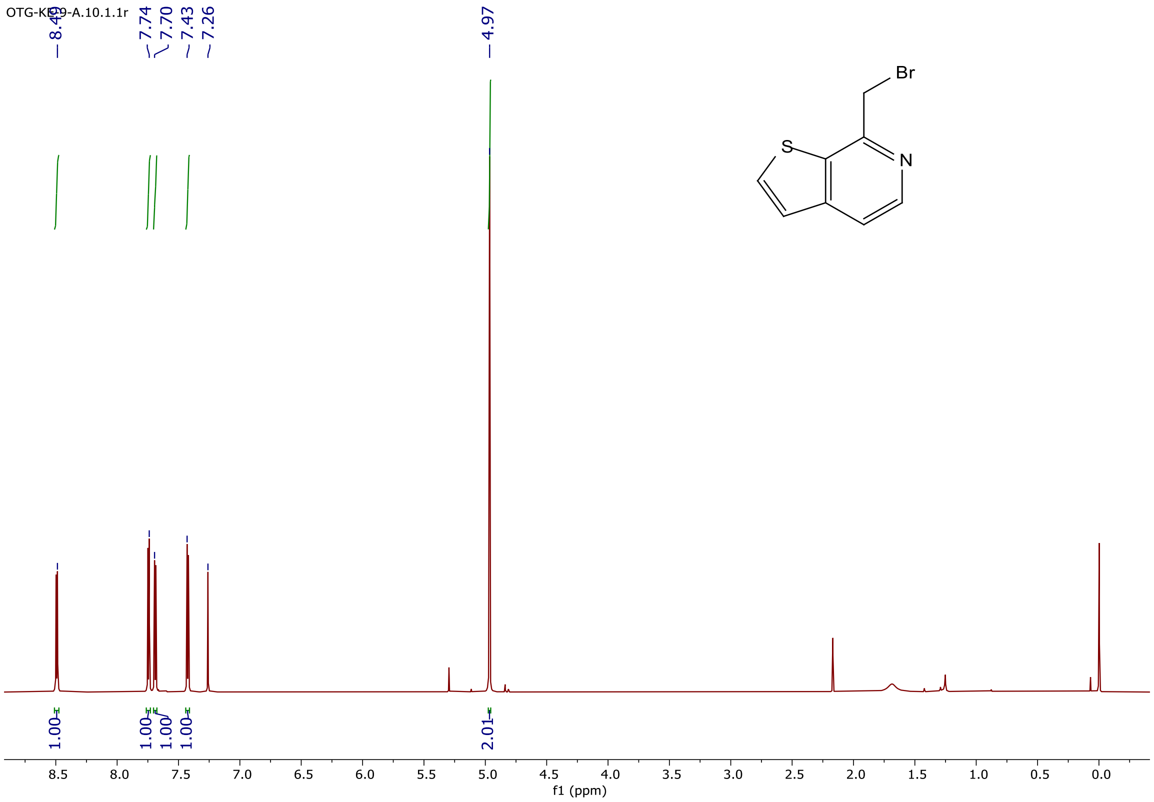

3a, ^{13}C (APT), 126 MHz, $CDCl_3$

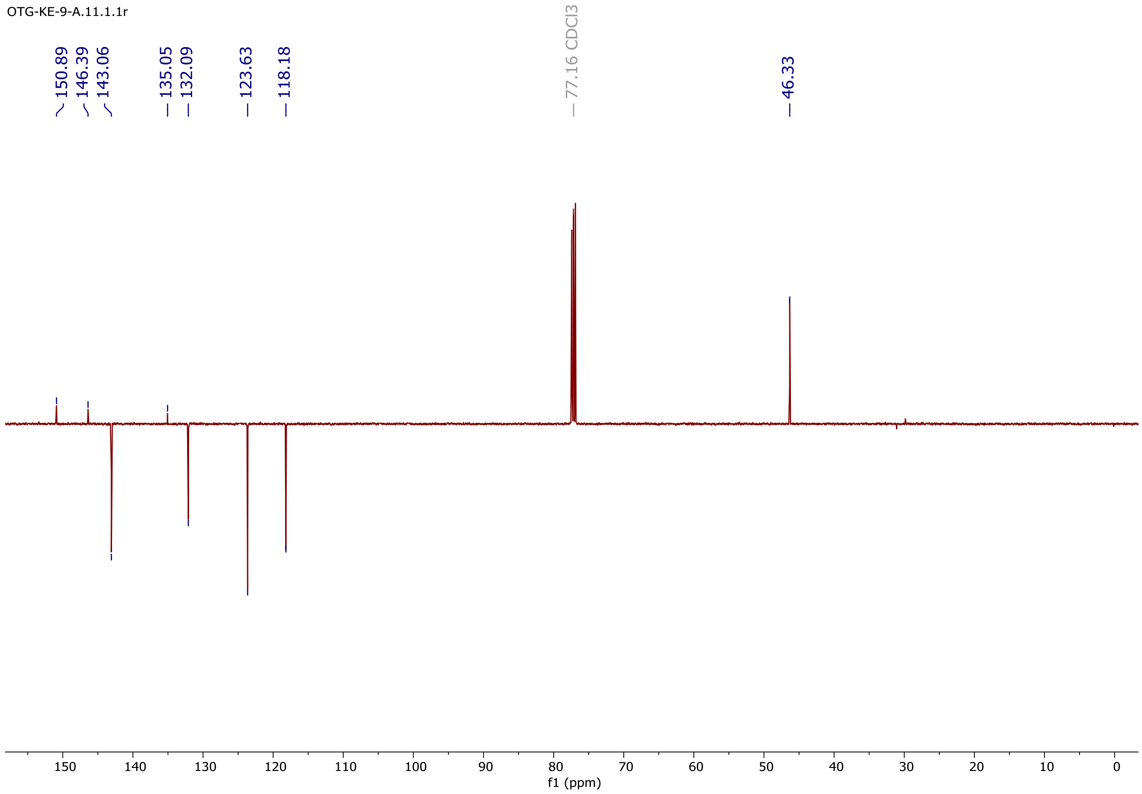

3b, ^{1}H, 500 MHz, $CDCl_3$

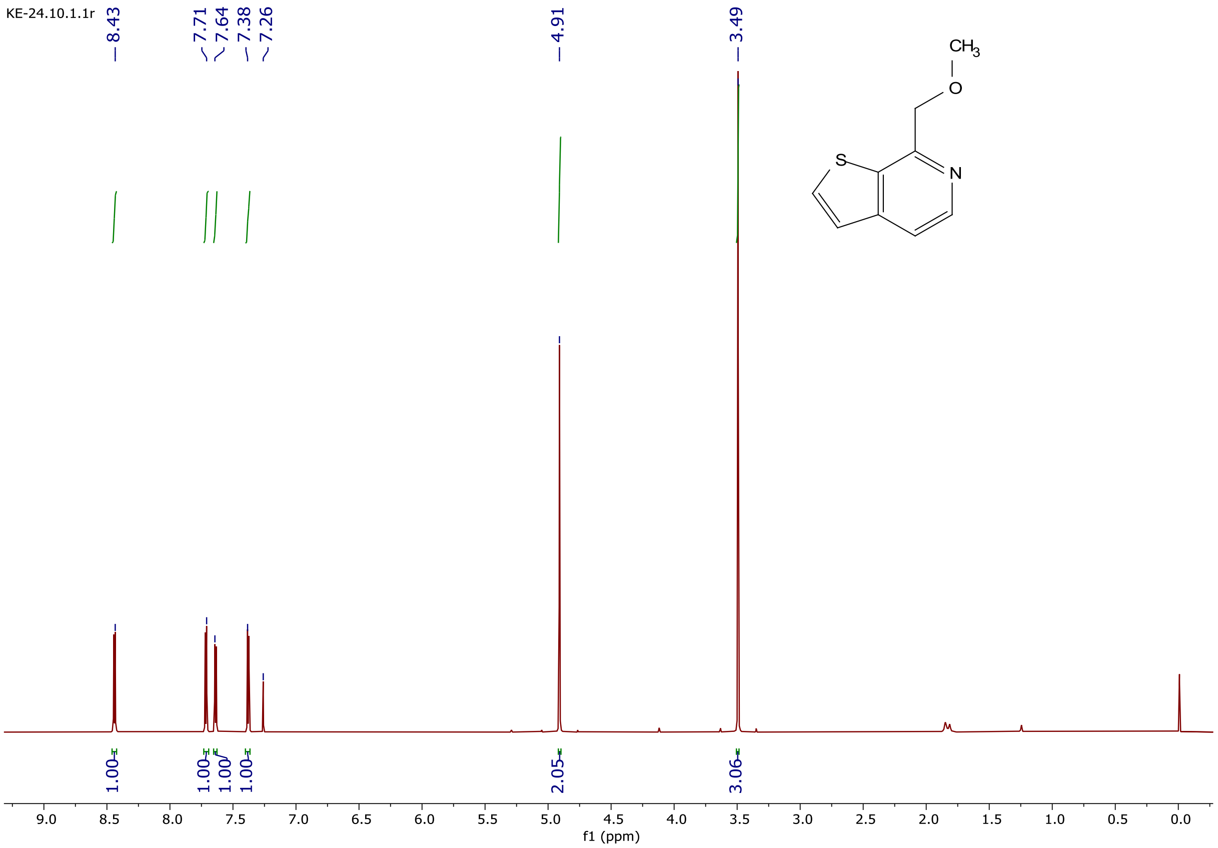

3b, ^{13}C, 126 MHz, $CDCl_3$

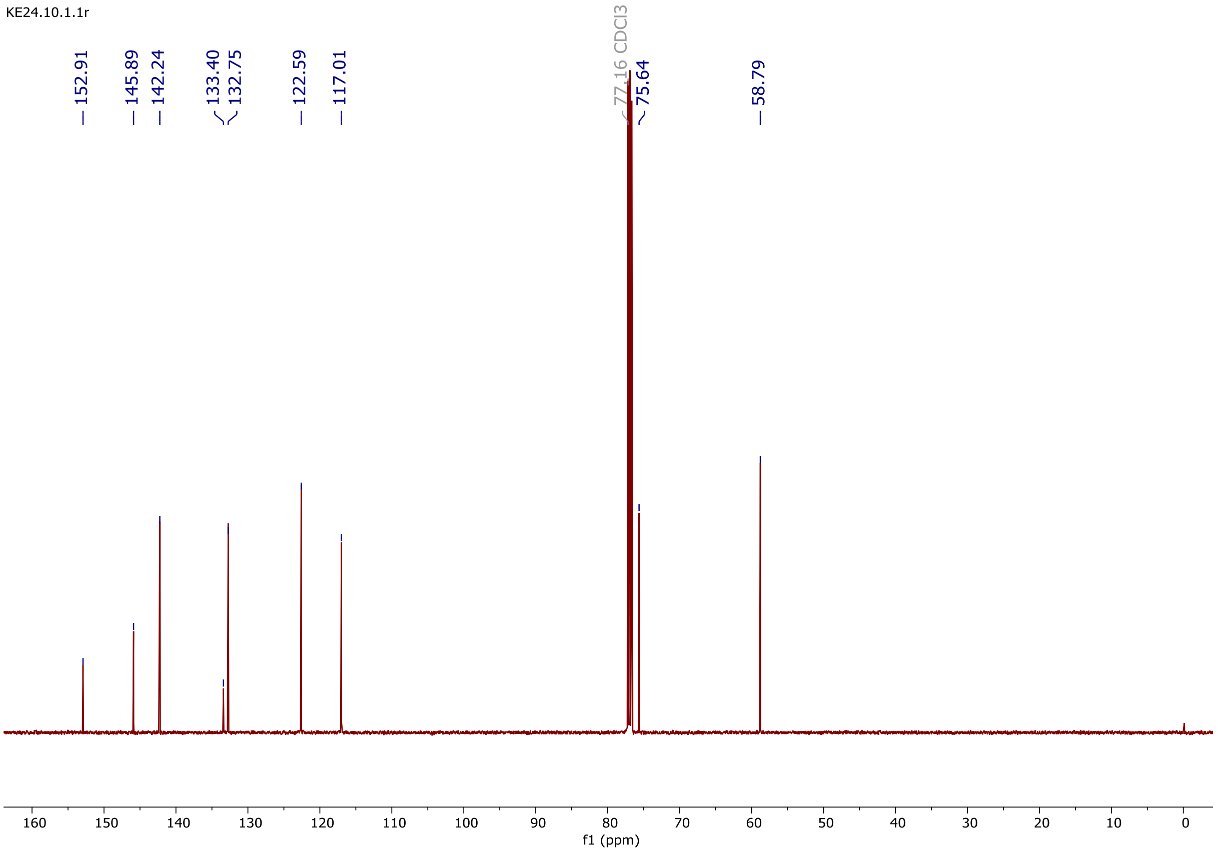

3c, ^{1}H, 500 MHz, $CDCl_3$

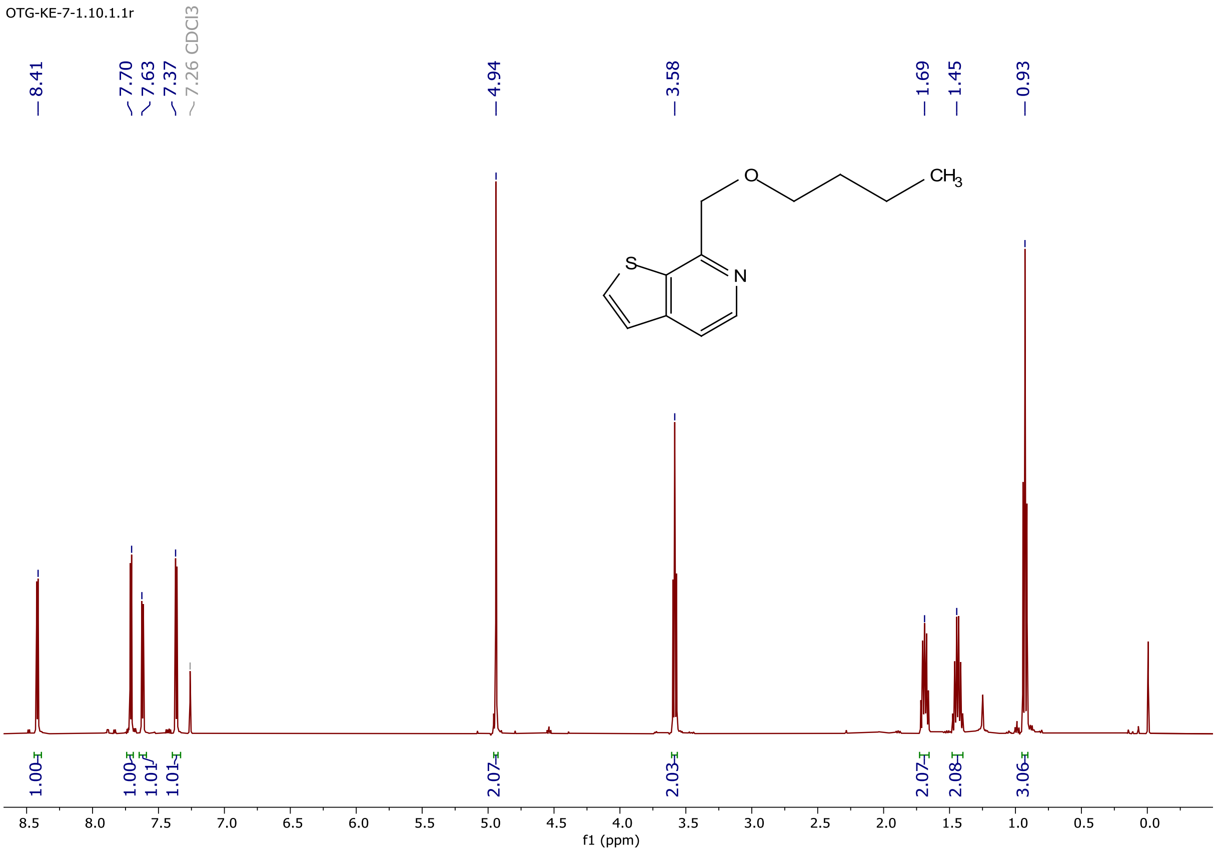

3c, ^{13}C (APT), 126 MHz, $CDCl_3$

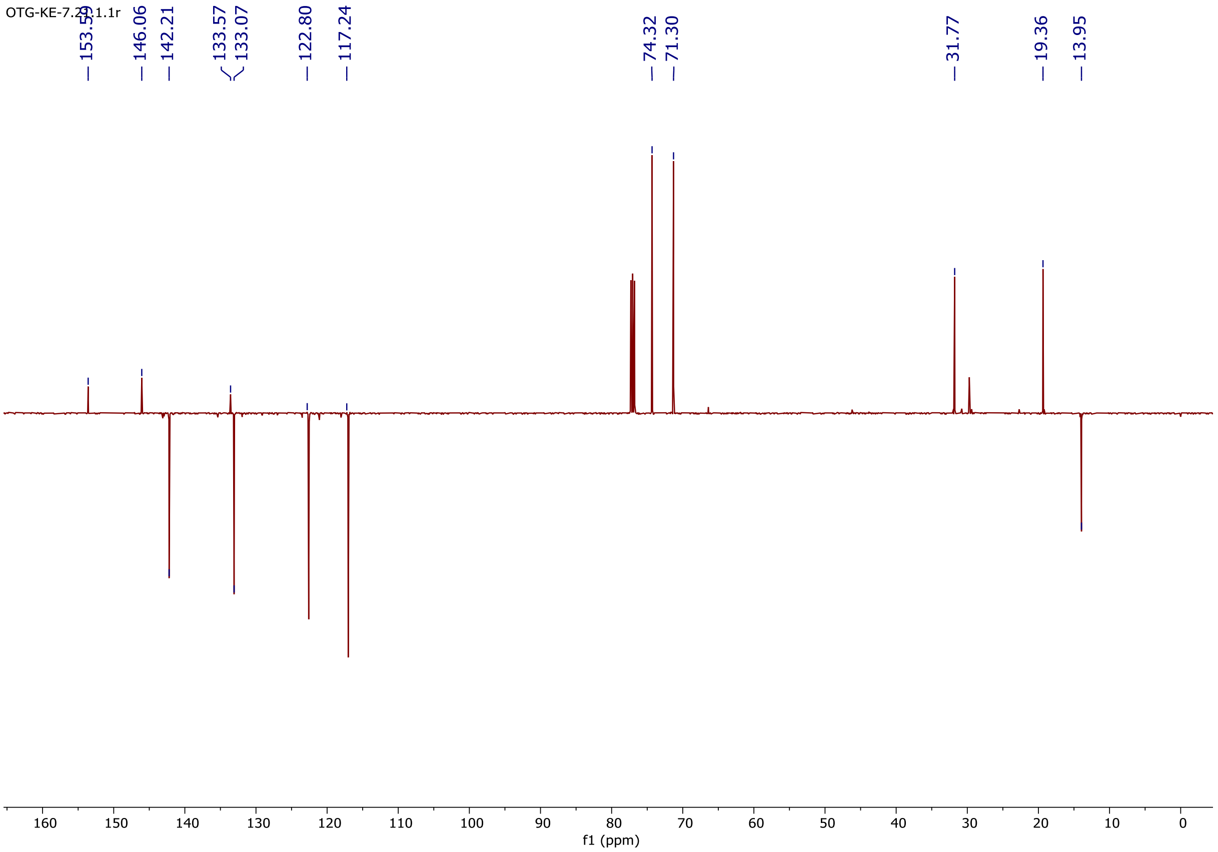

3d, ^{1}H, 500 MHz, $CDCl_3$

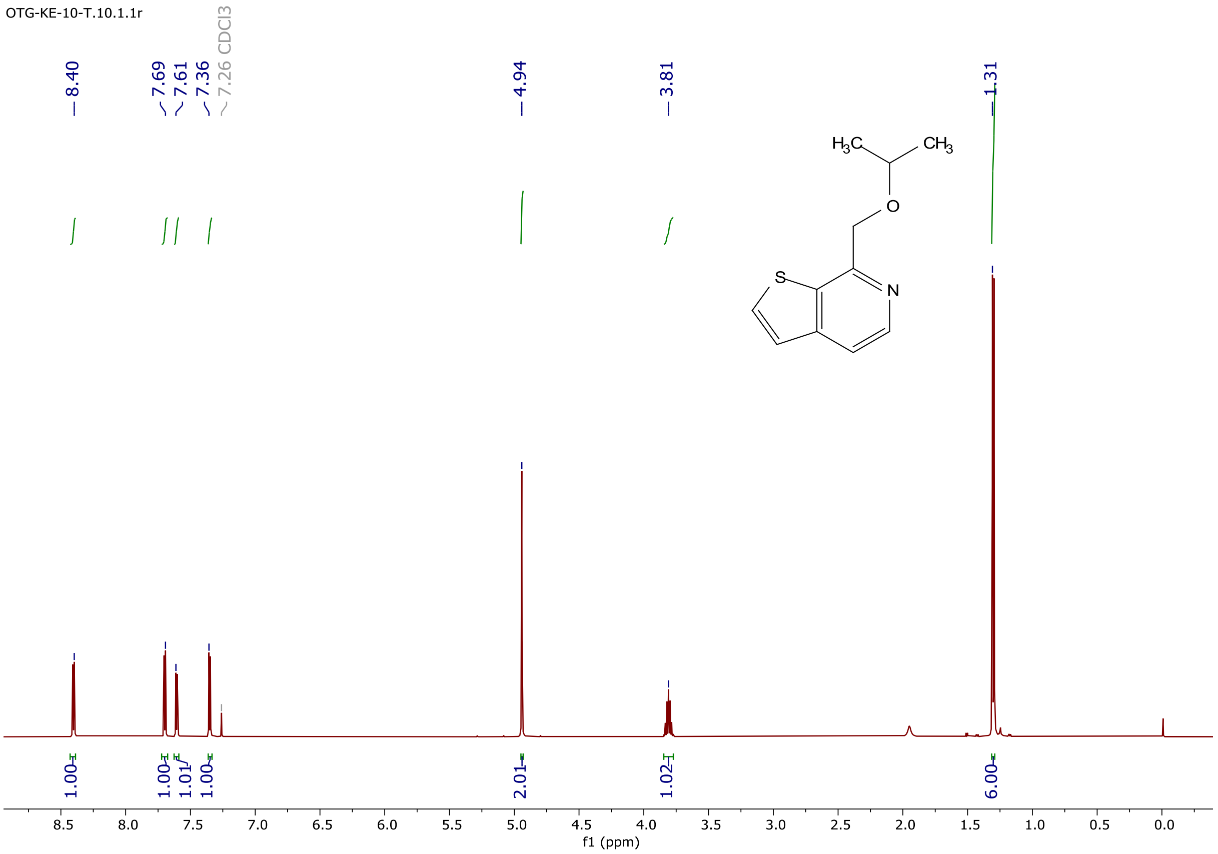

3d, ^{13}C (APT), 126 MHz, $CDCl_3$

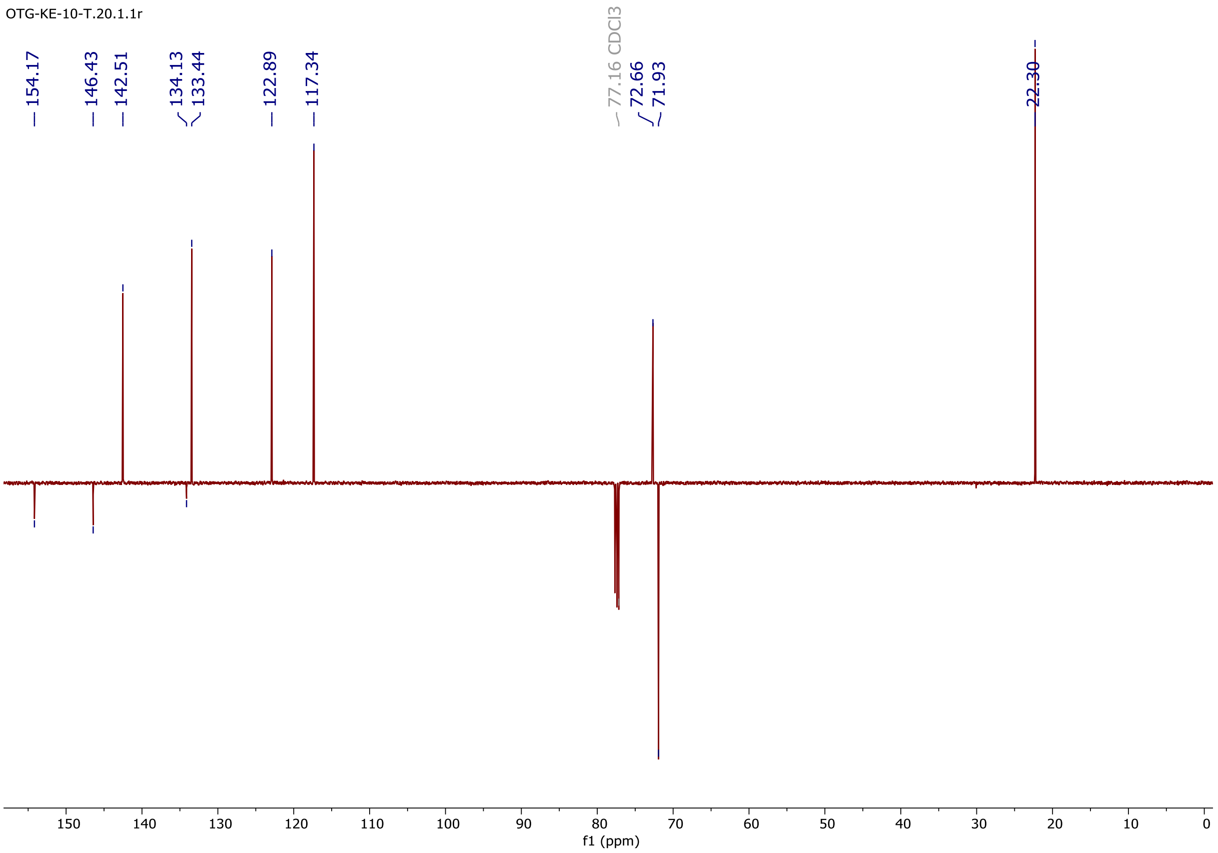

3e, ^{1}H, 500 MHz, $CDCl_3$

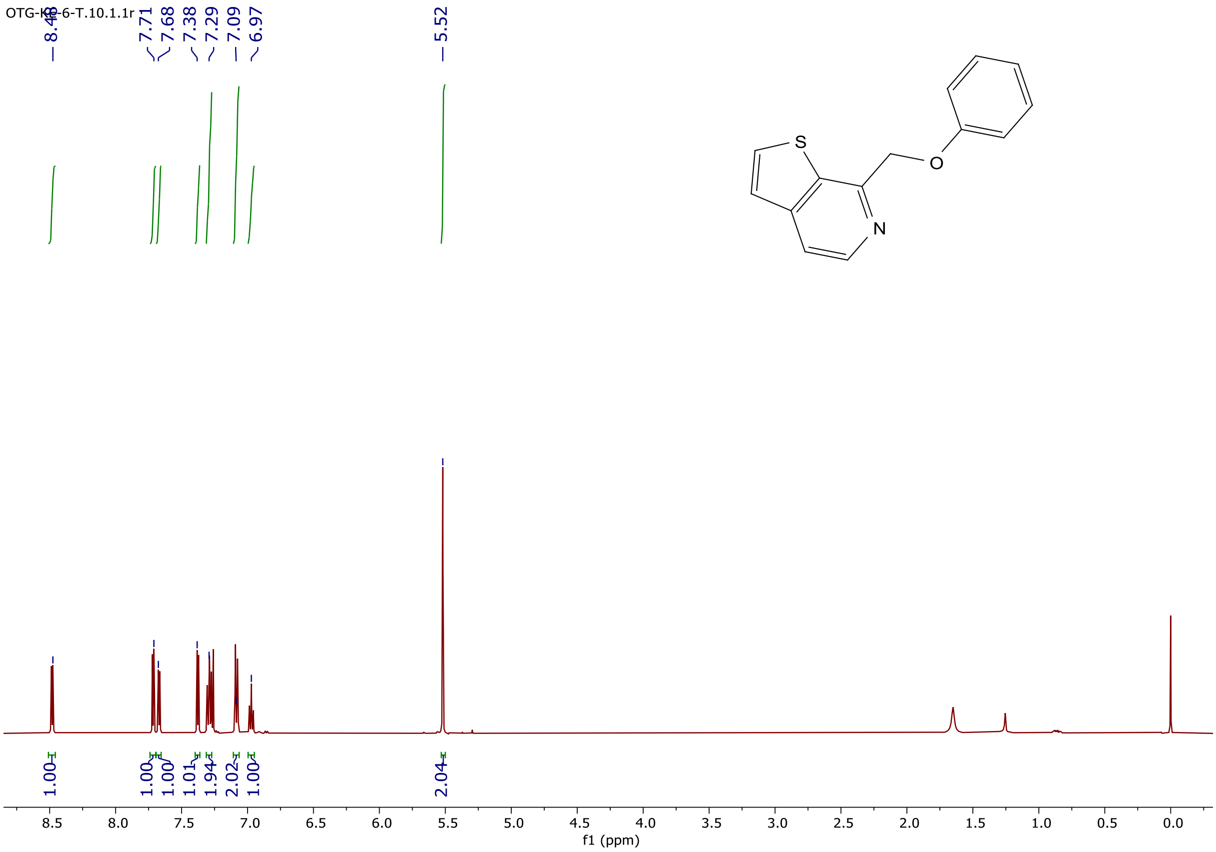

3e, ^{13}C (APT), 126 MHz, $CDCl_3$

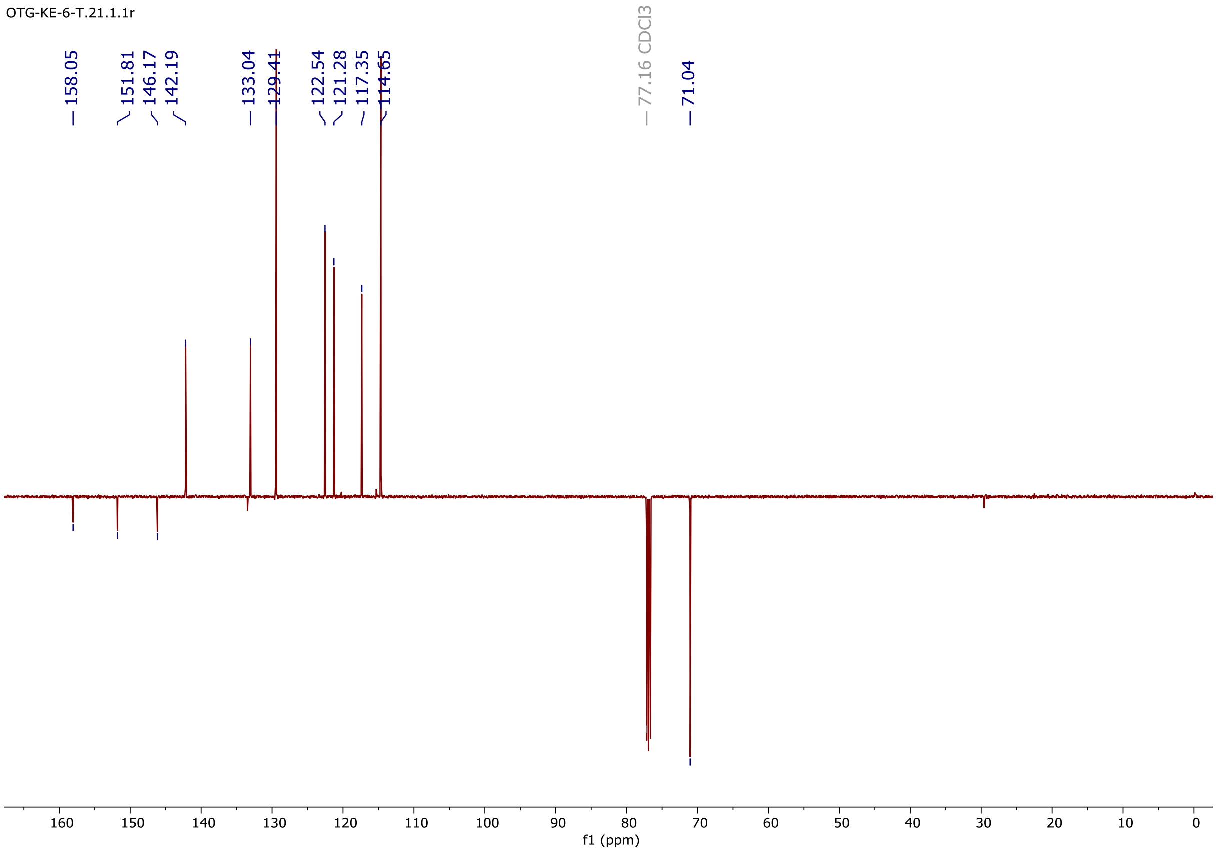

3f, ^{1}H, 500 MHz, $CDCl_3$

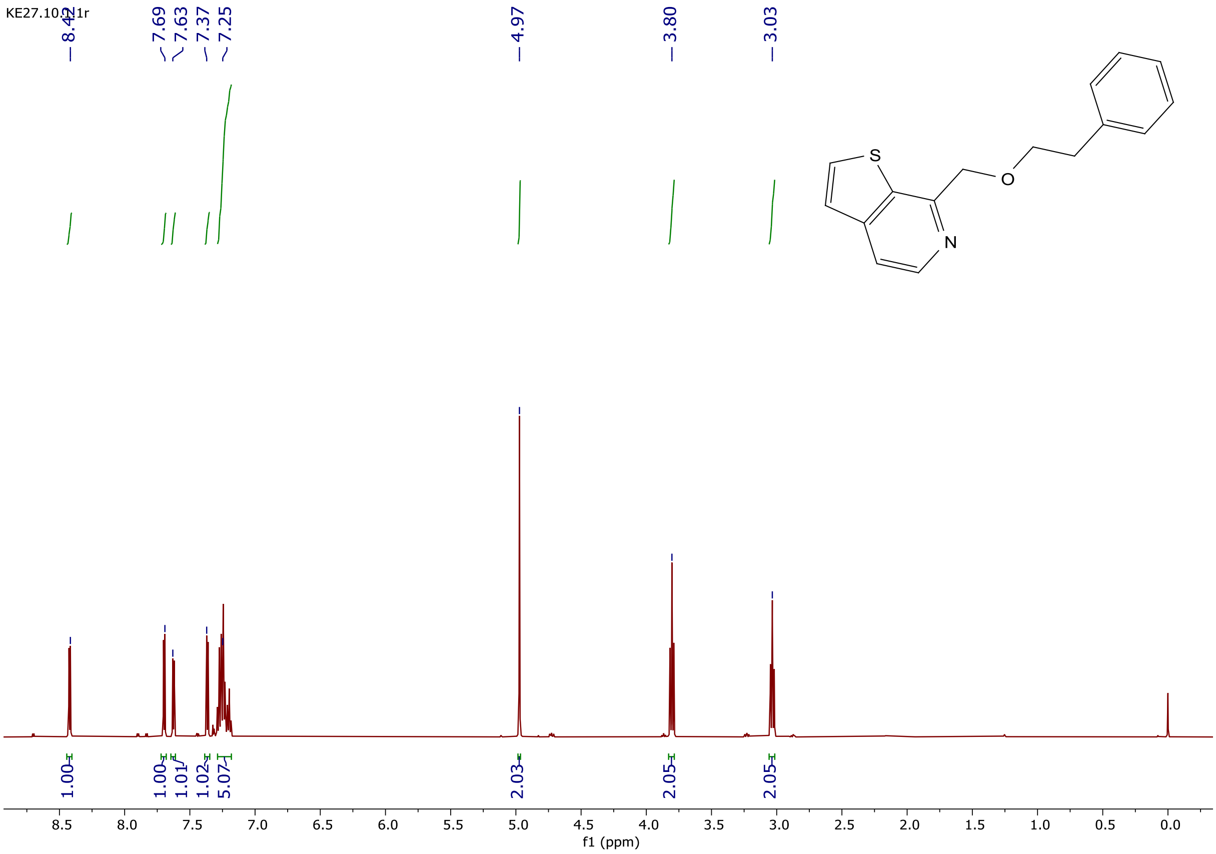

3f, ^{13}C (APT), 126 MHz, $CDCl_3$

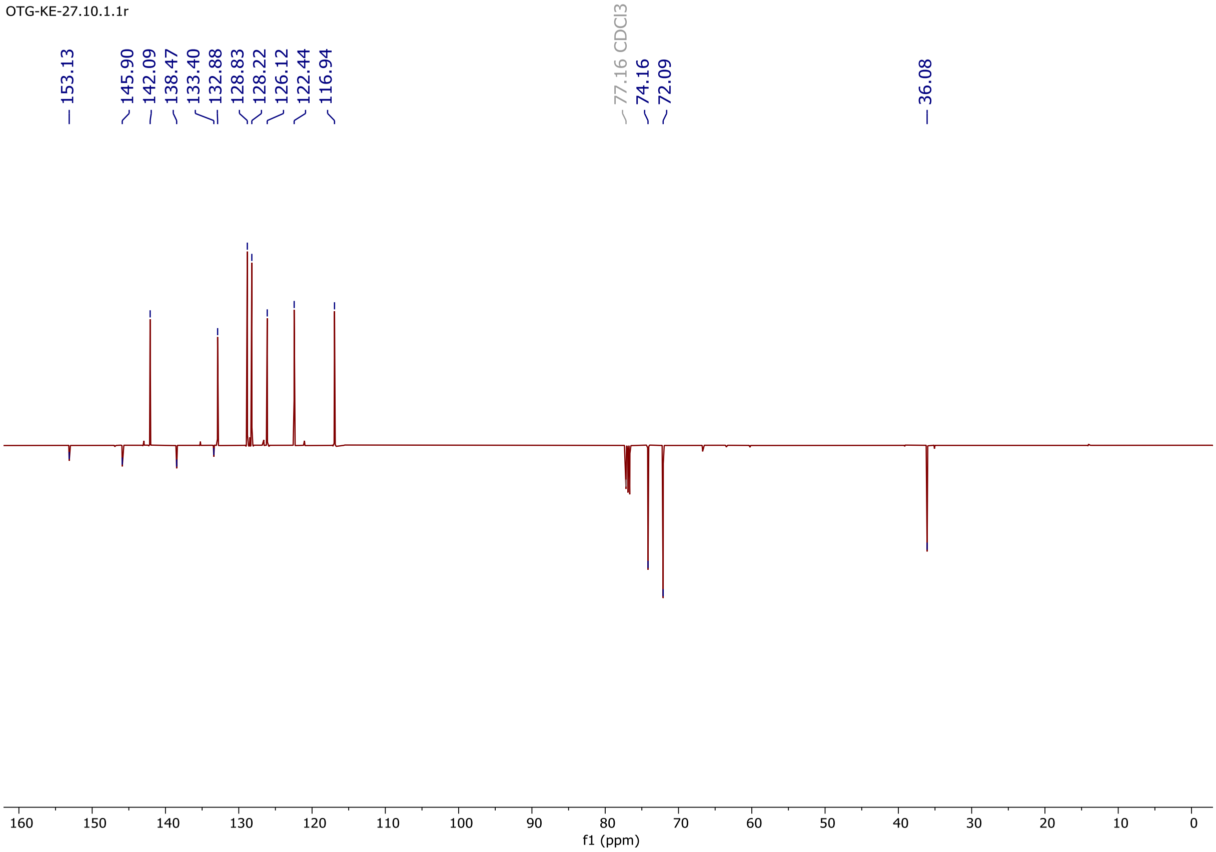

3g, ^{1}H, 500 MHz, $CDCl_3$

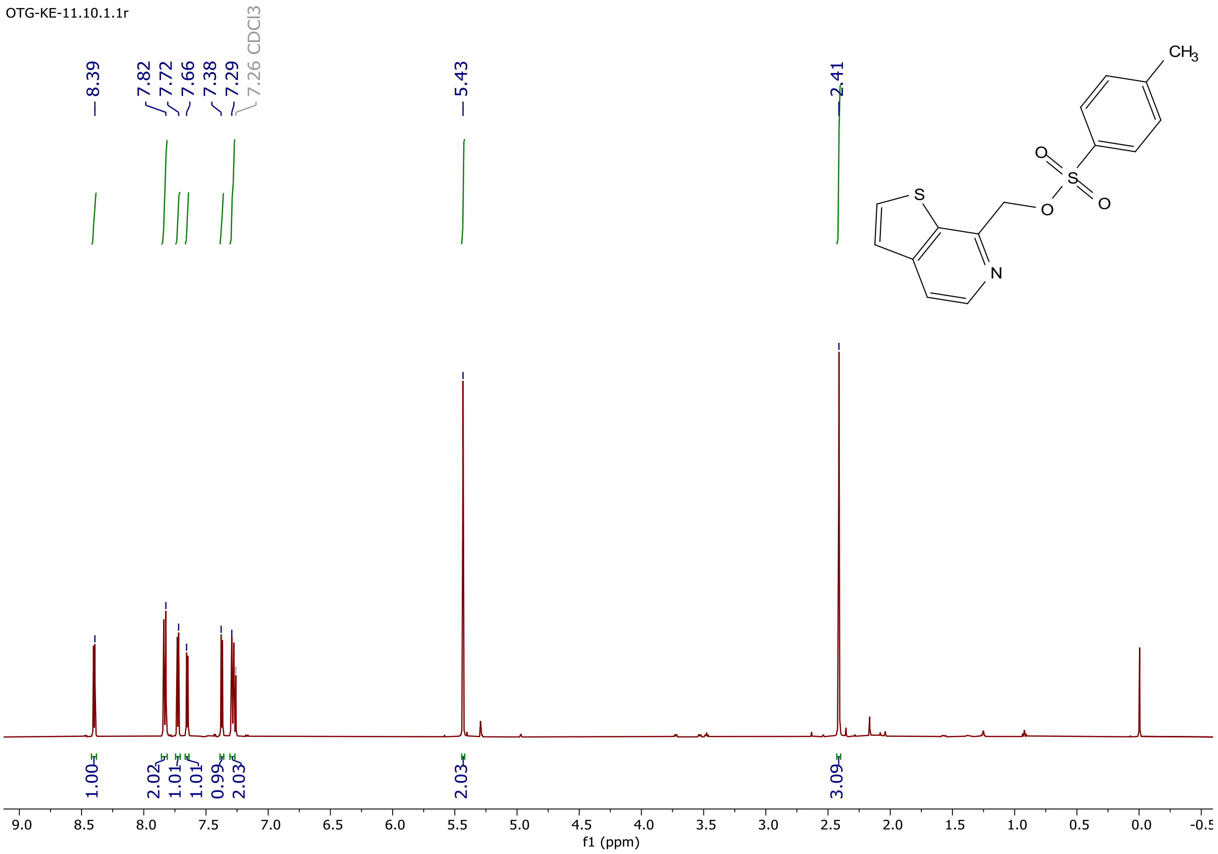

3g, ^{13}C (APT), 126 MHz, $CDCl_3$

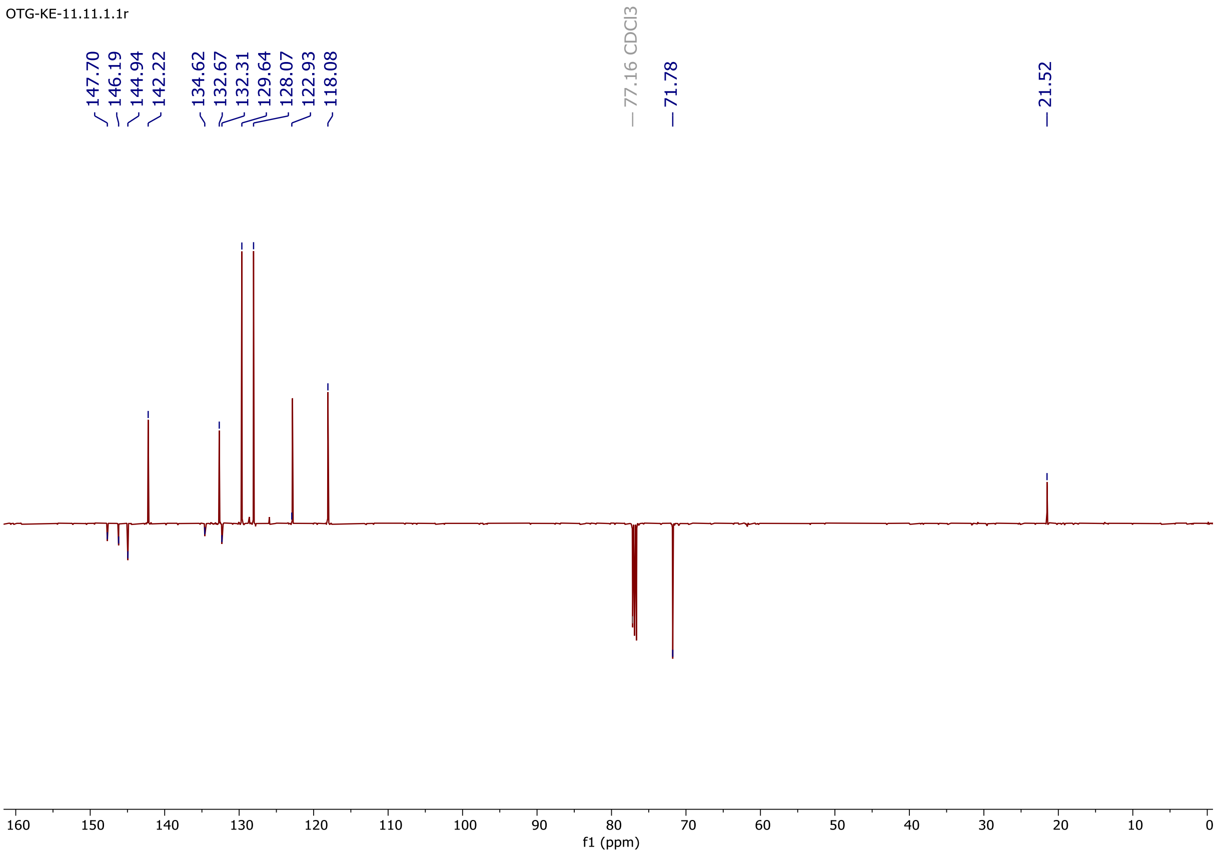

3h, ^{1}H, 500 MHz, $CDCl_3$

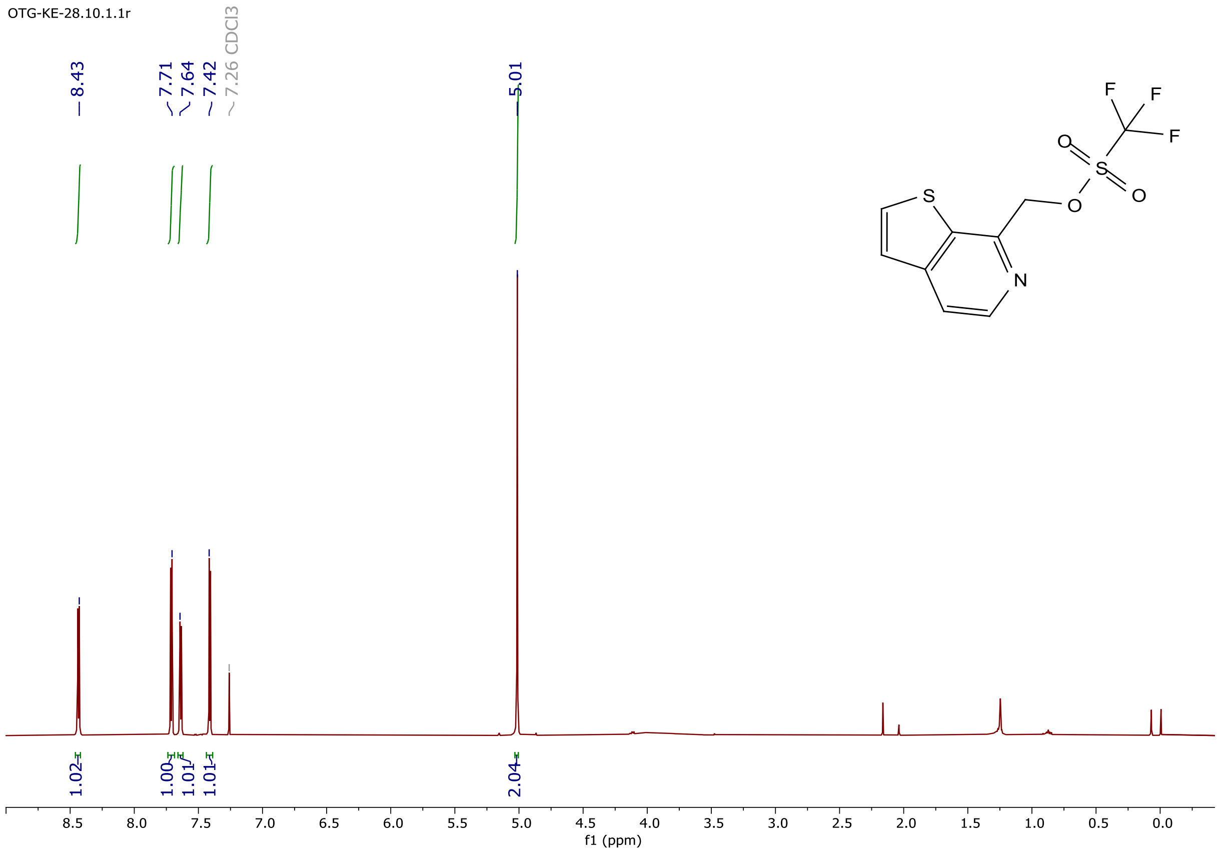

3h, ^{13}C (APT), 126 MHz, $CDCl_3$

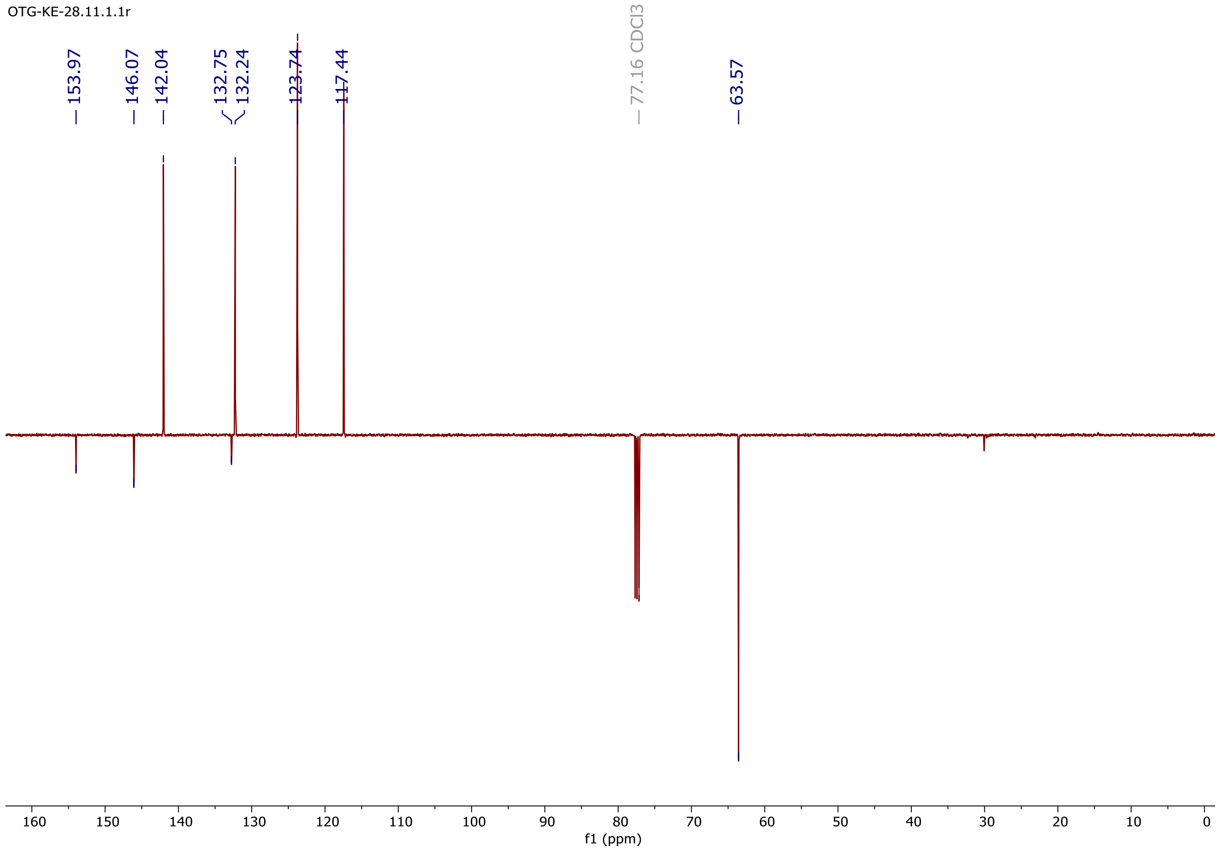

4a, ^{1}H, 500 MHz, $CDCl_3$

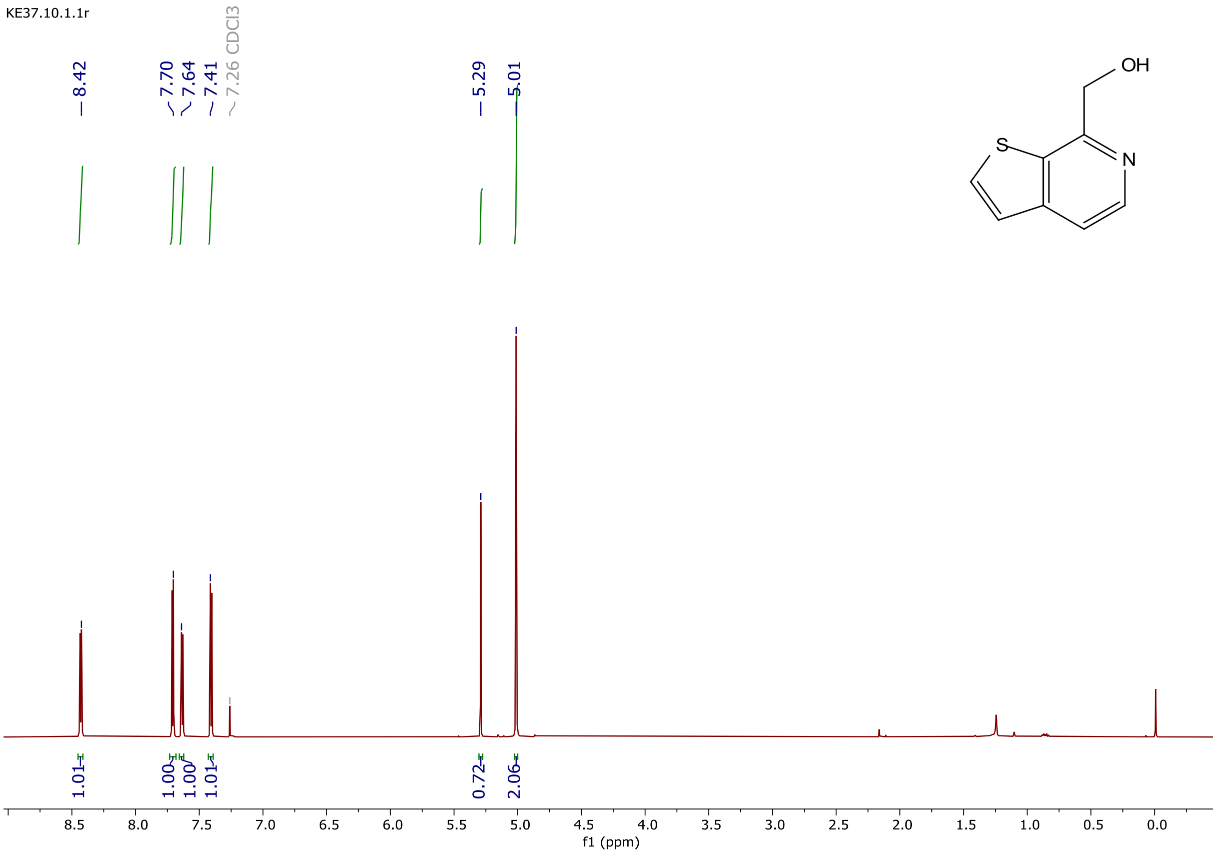

4a, ^{13}C (APT), 126 MHz, $CDCl_3$

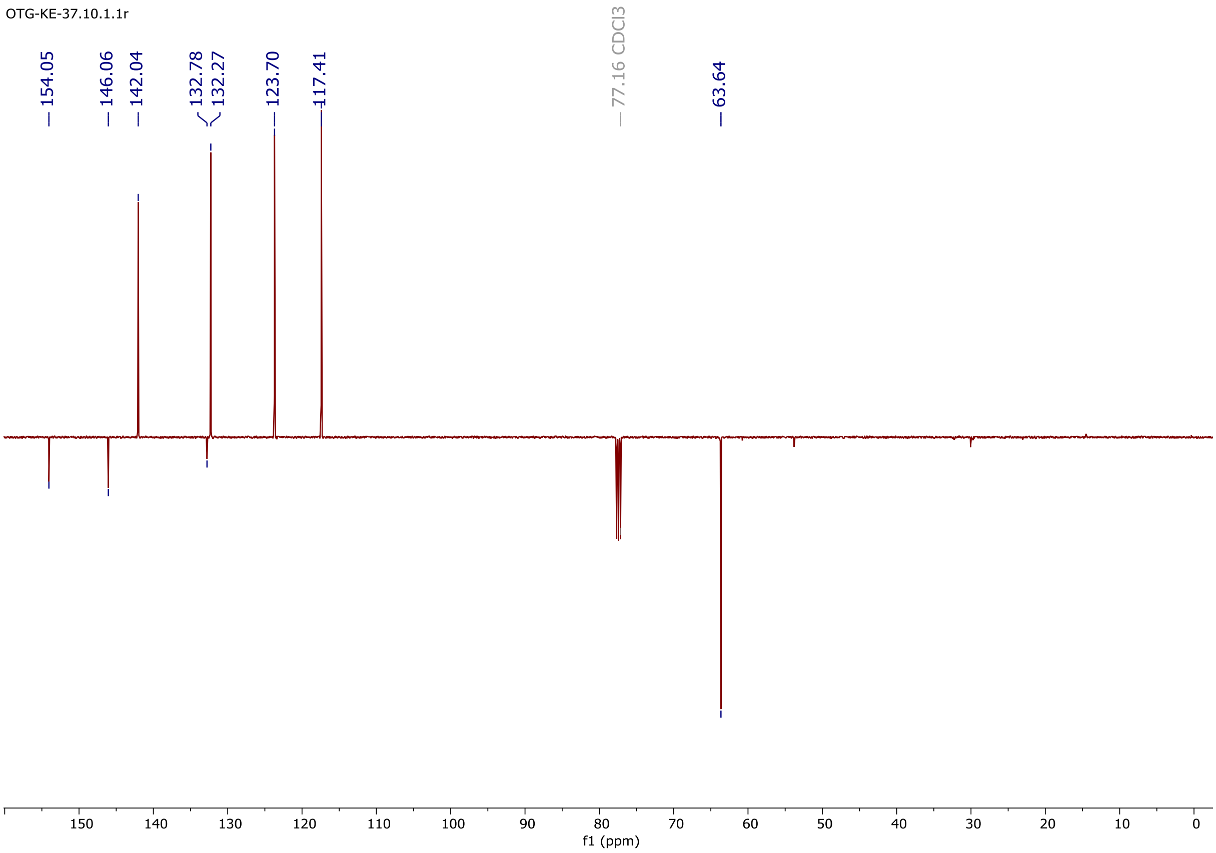

4b, ^{1}H, 500 MHz, $CDCl_3$

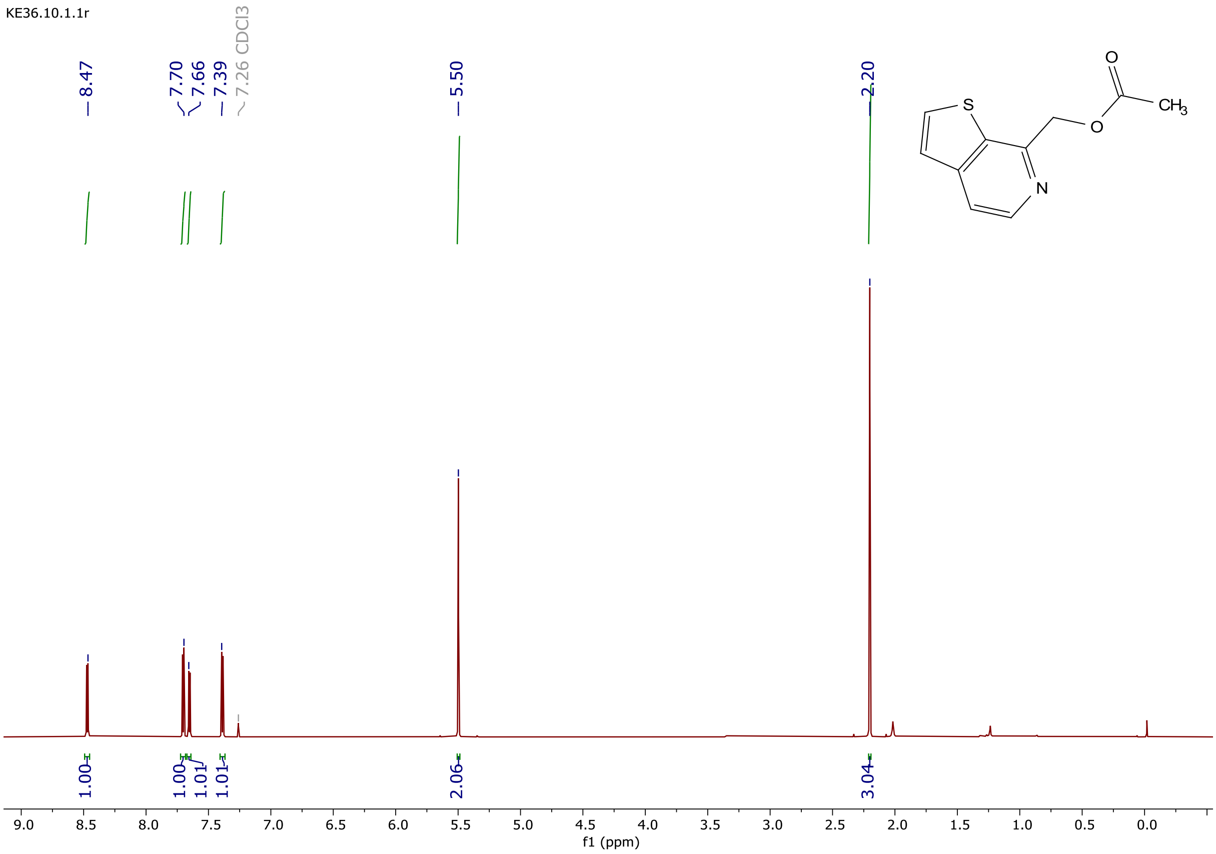

4b, ^{13}C (APT), 126 MHz, $CDCl_3$

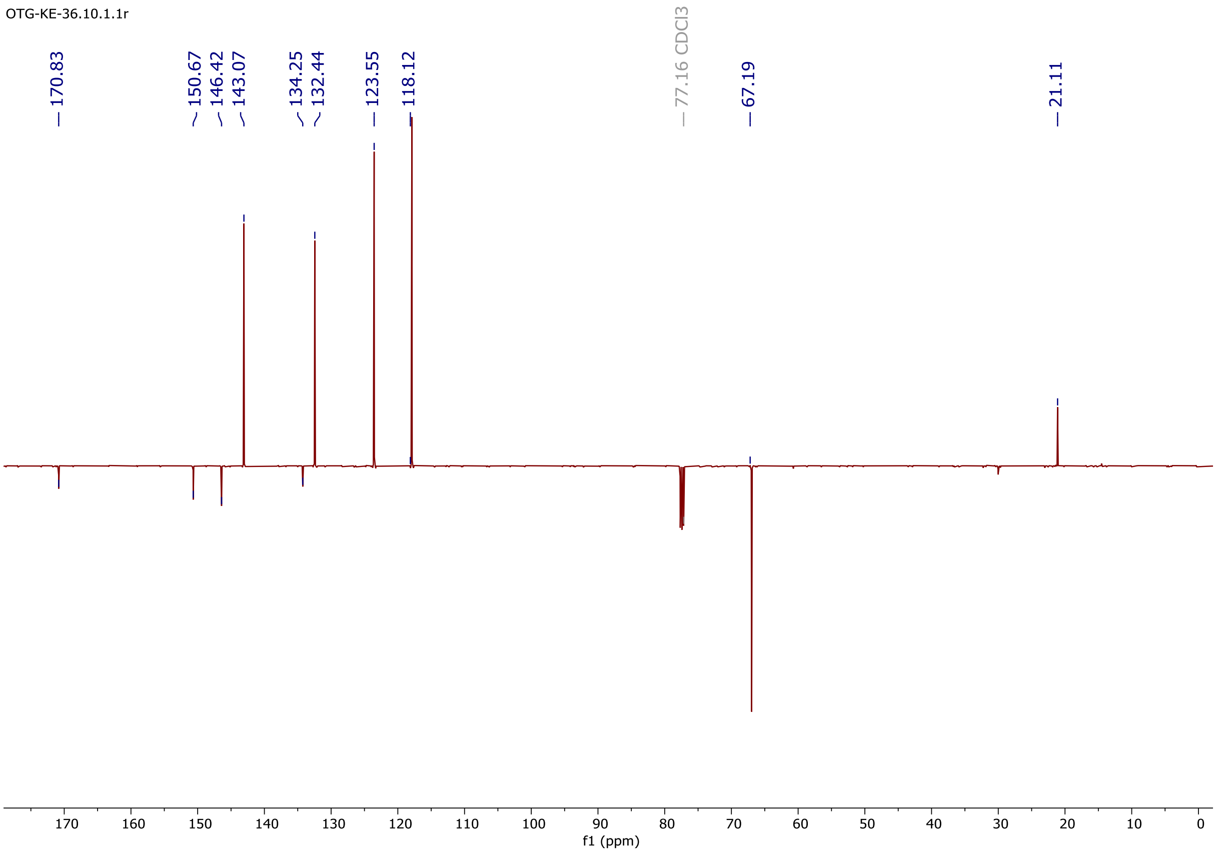

4c, ^{1}H, 500 MHz, $CDCl_3$

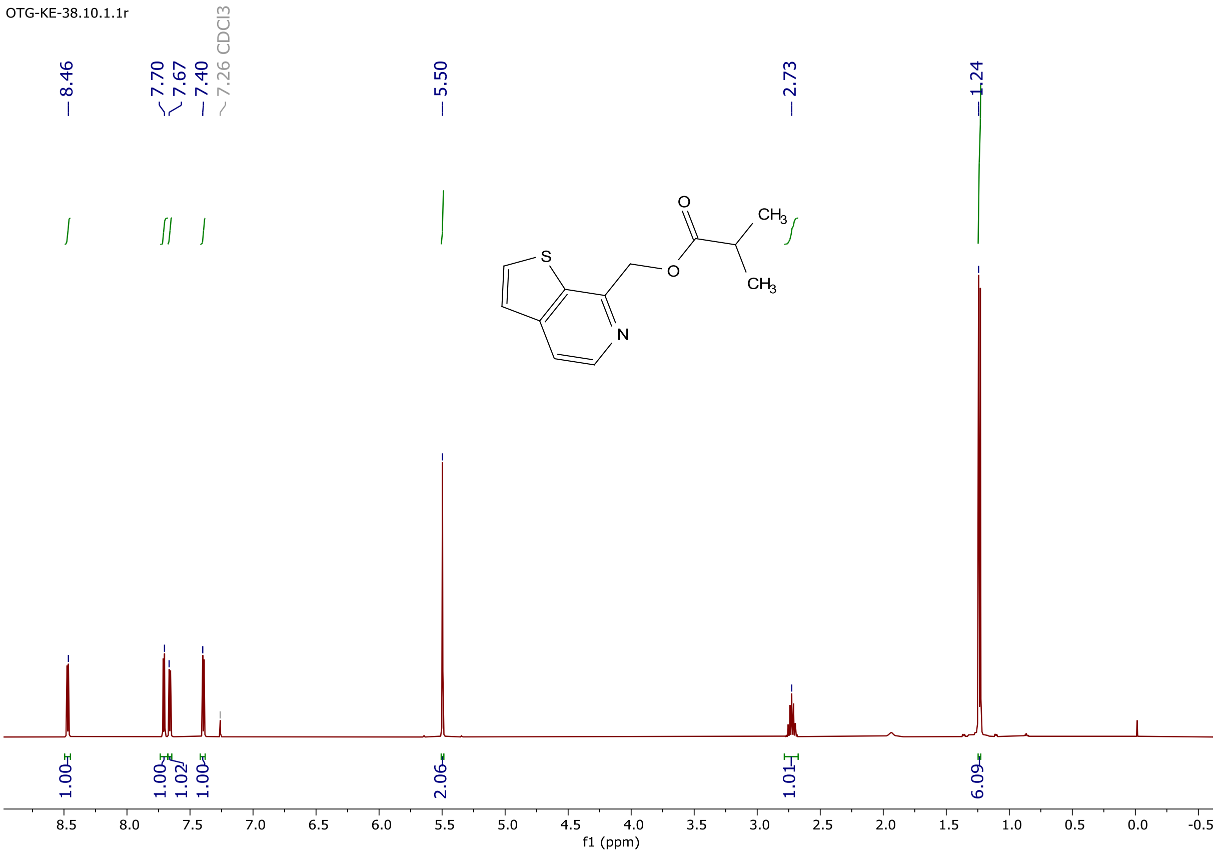

4c, ^{13}C (APT), 126 MHz, $CDCl_3$

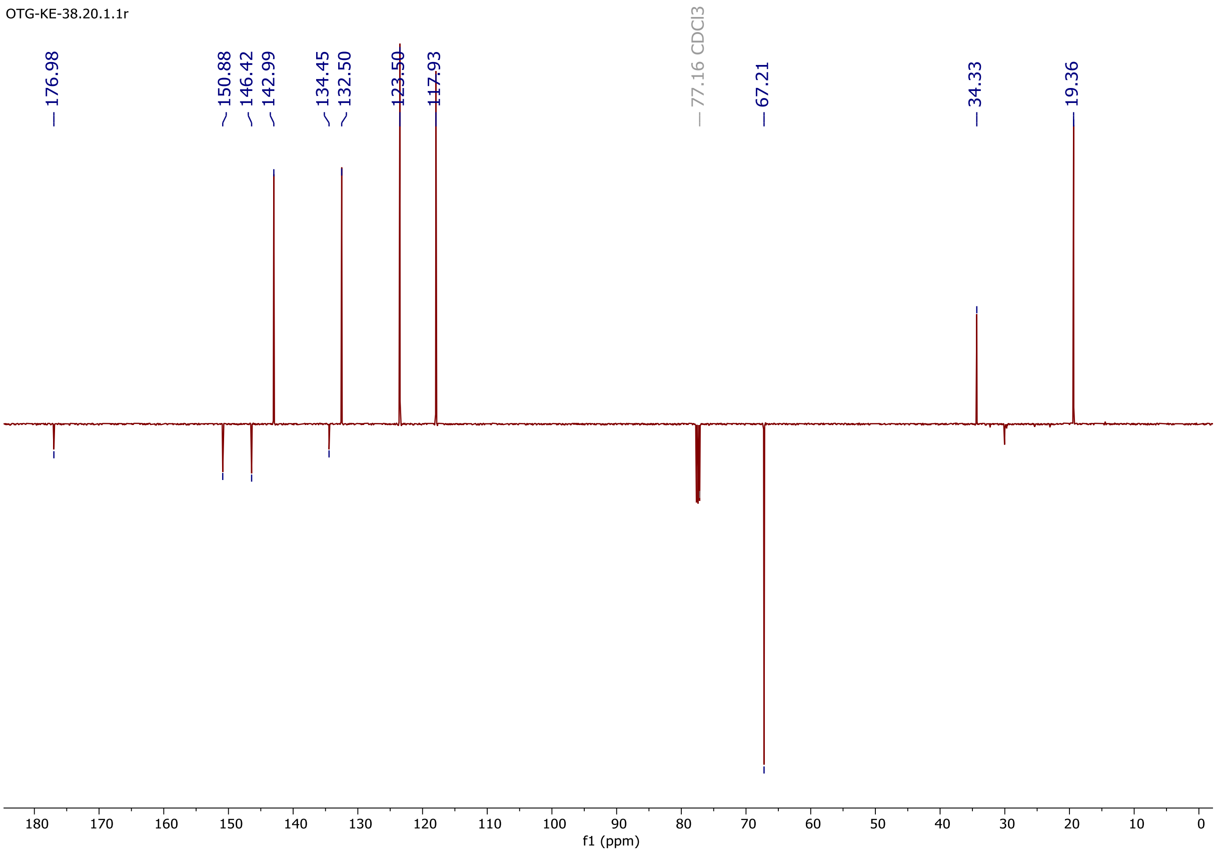

4d, ^{1}H, 500 MHz, $CDCl_3$

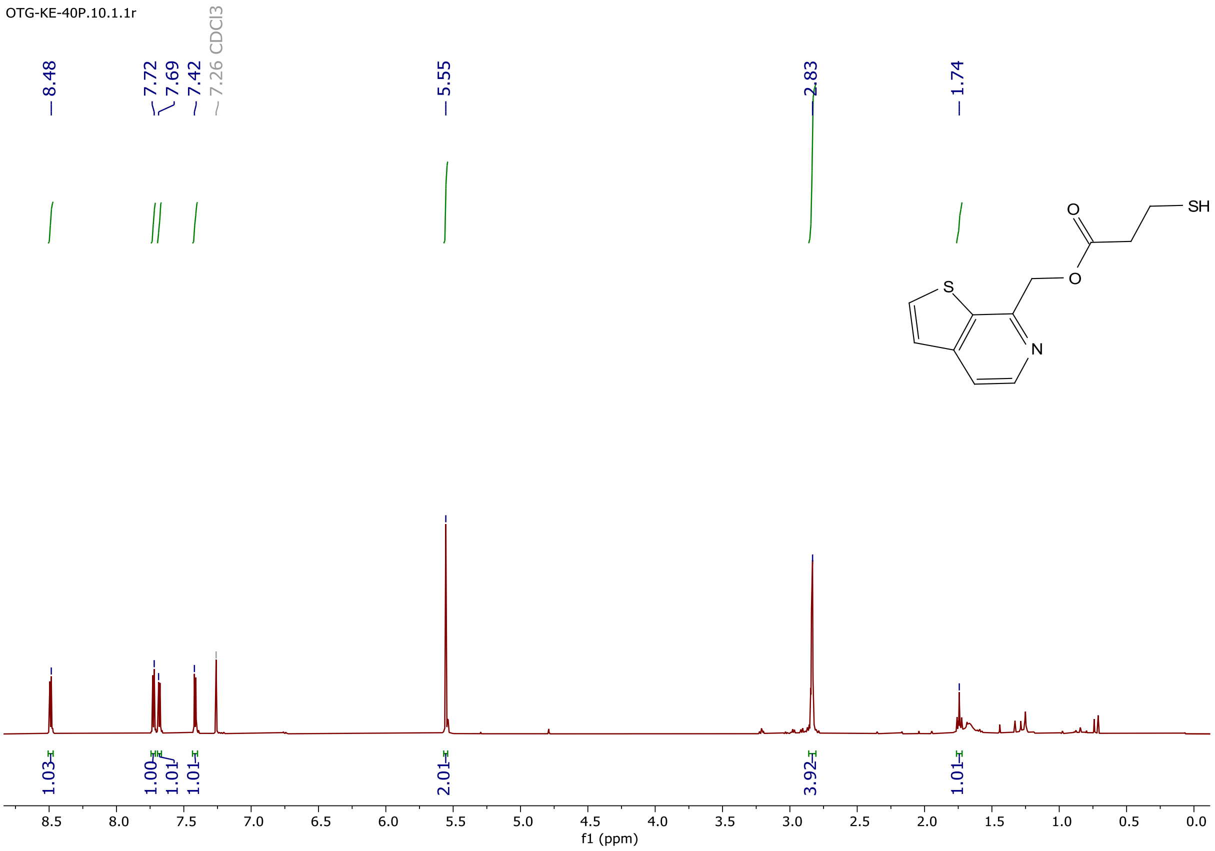

4d, ^{13}C (APT), 126 MHz, $CDCl_3$

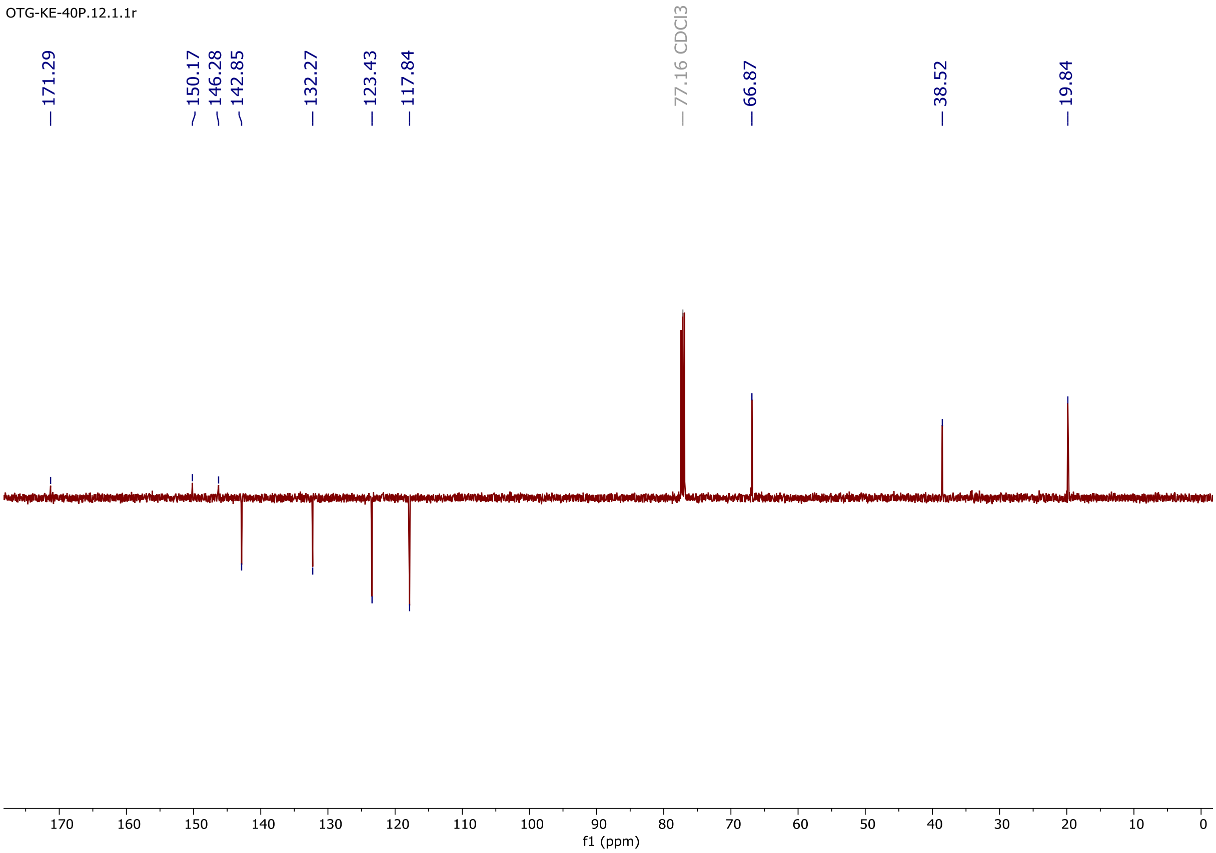

4e, ^{1}H, 500 MHz, $CDCl_3$

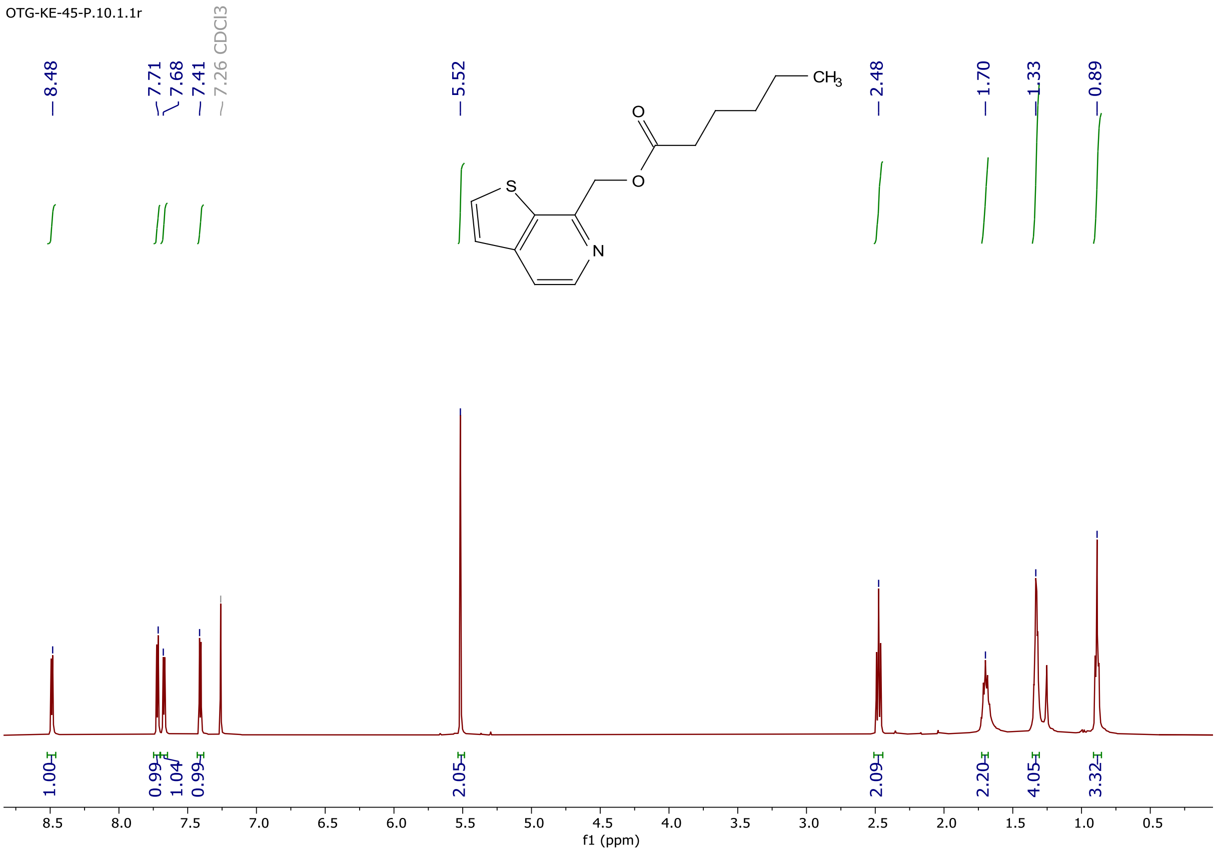

4e, ^{13}C (APT), 126 MHz, $CDCl_3$

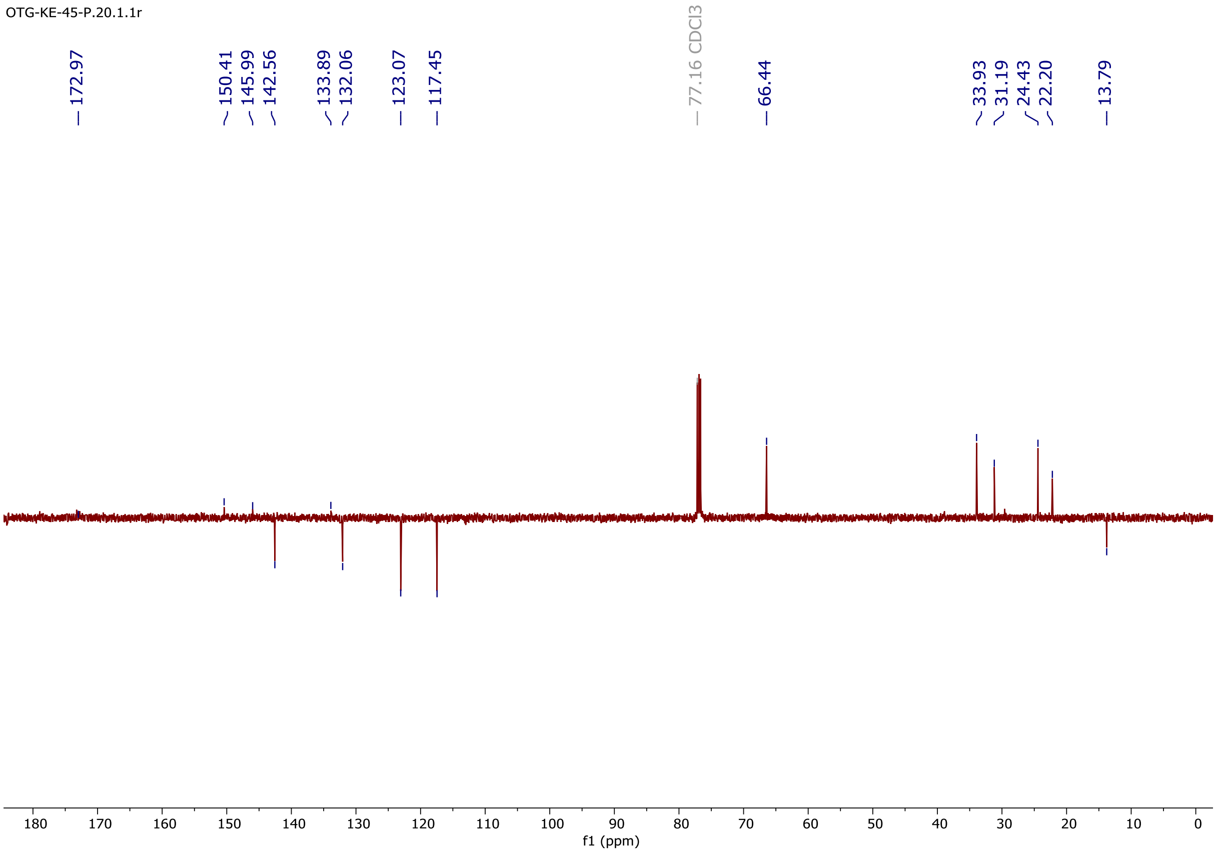

4f, ^{1}H, 500 MHz, $CDCl_3$

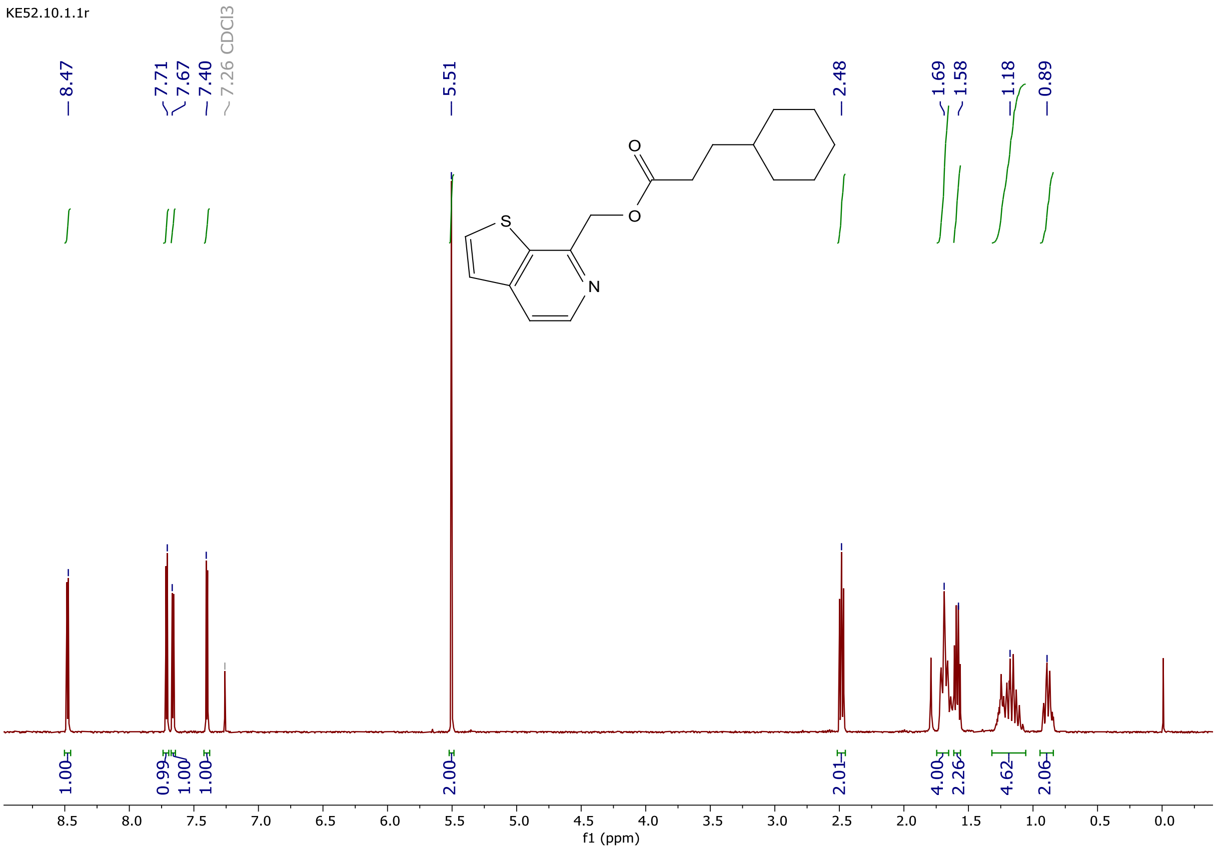

4f, ^{13}C (APT), 126 MHz, $CDCl_3$

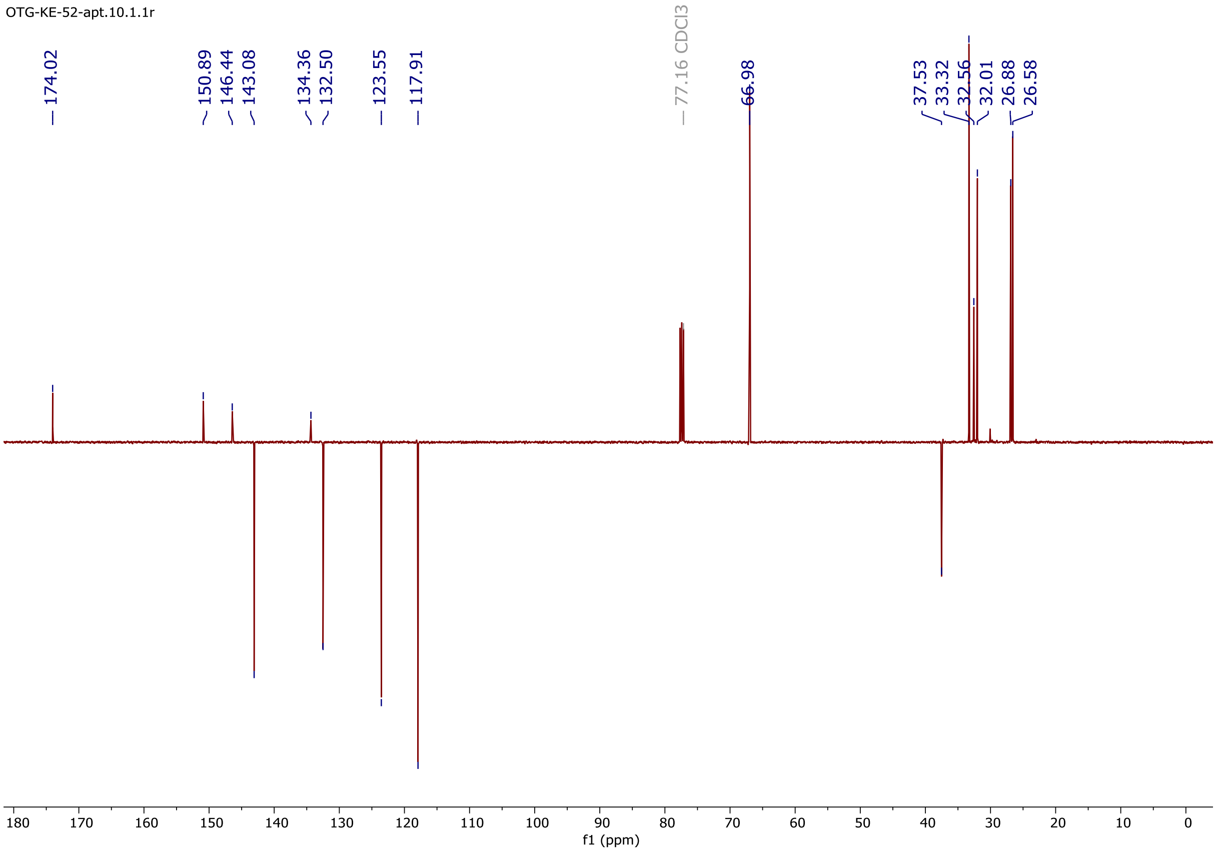

5a, ^{1}H, 500 MHz, $CDCl_3$

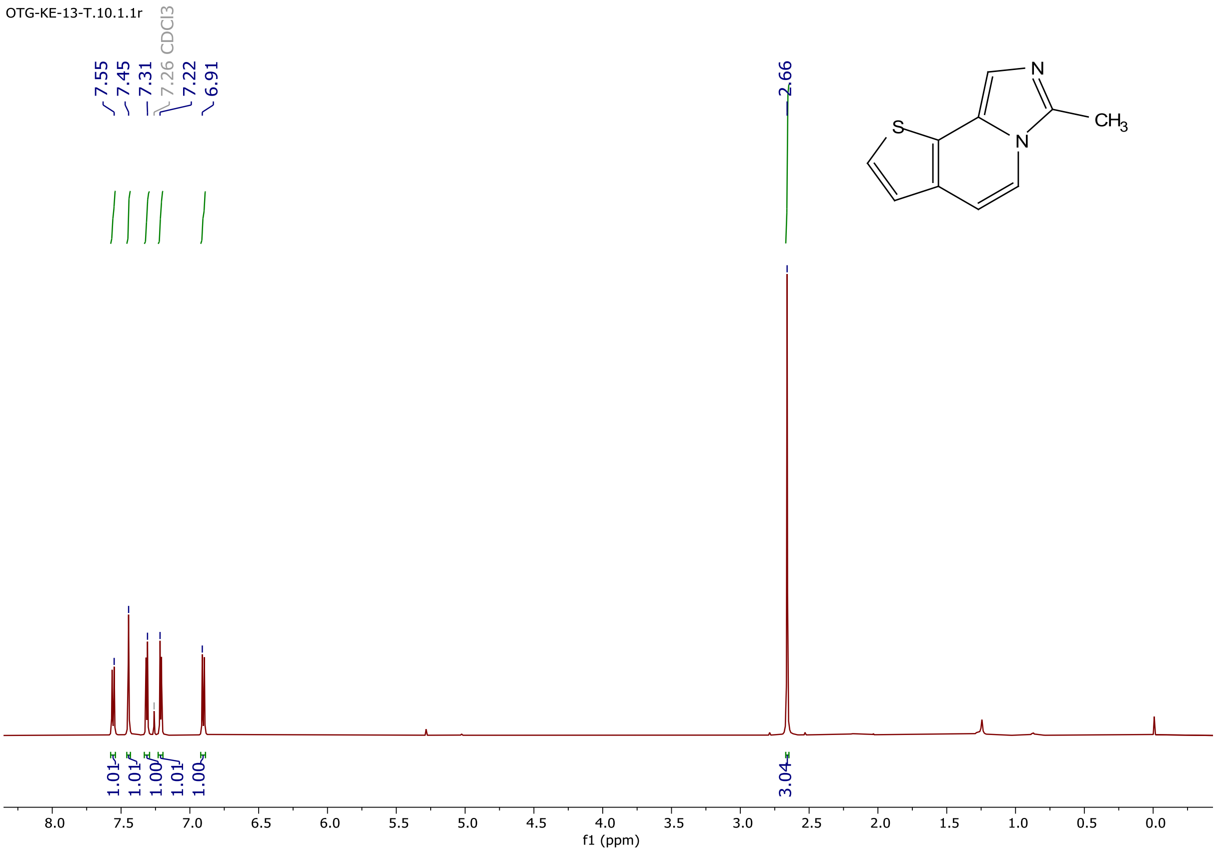

5a, ^{13}C (APT), 126 MHz, $CDCl_3$

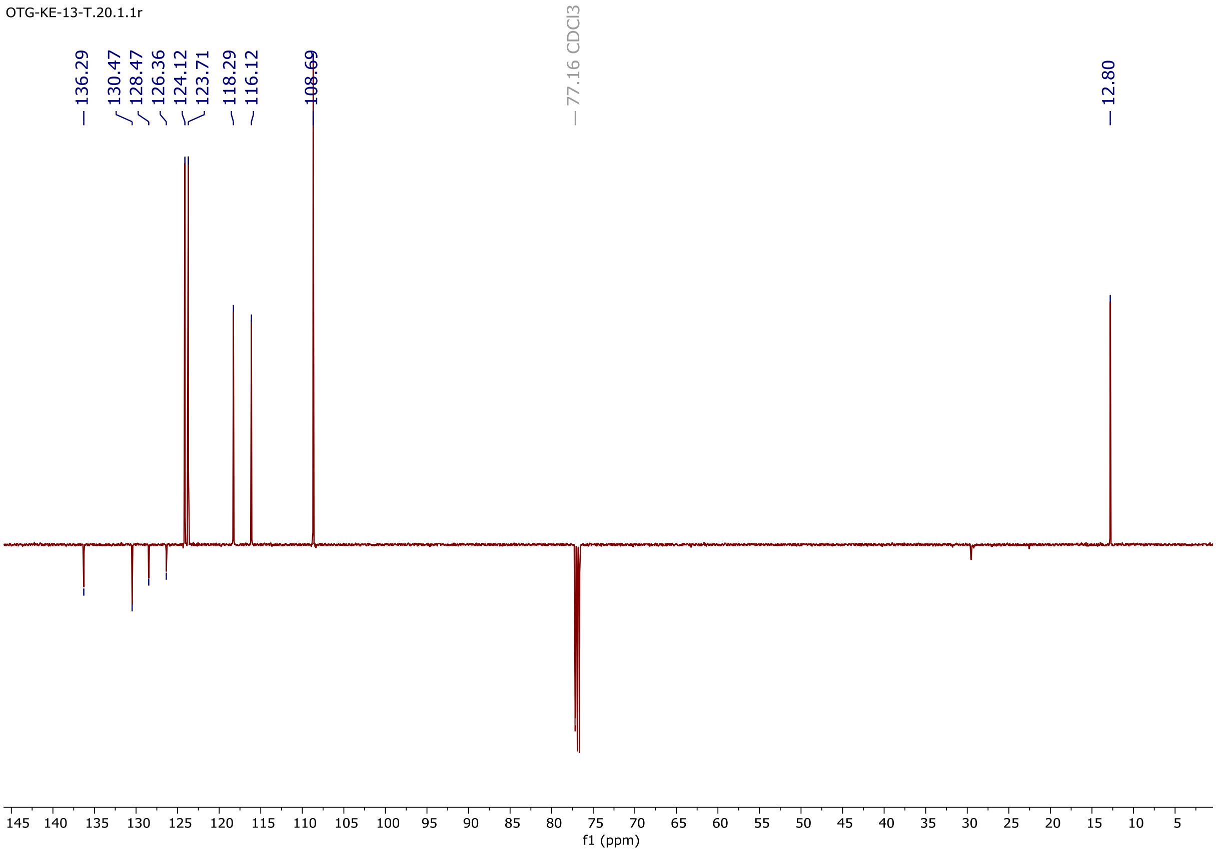

5b, ^{1}H, 500 MHz, $CDCl_3$

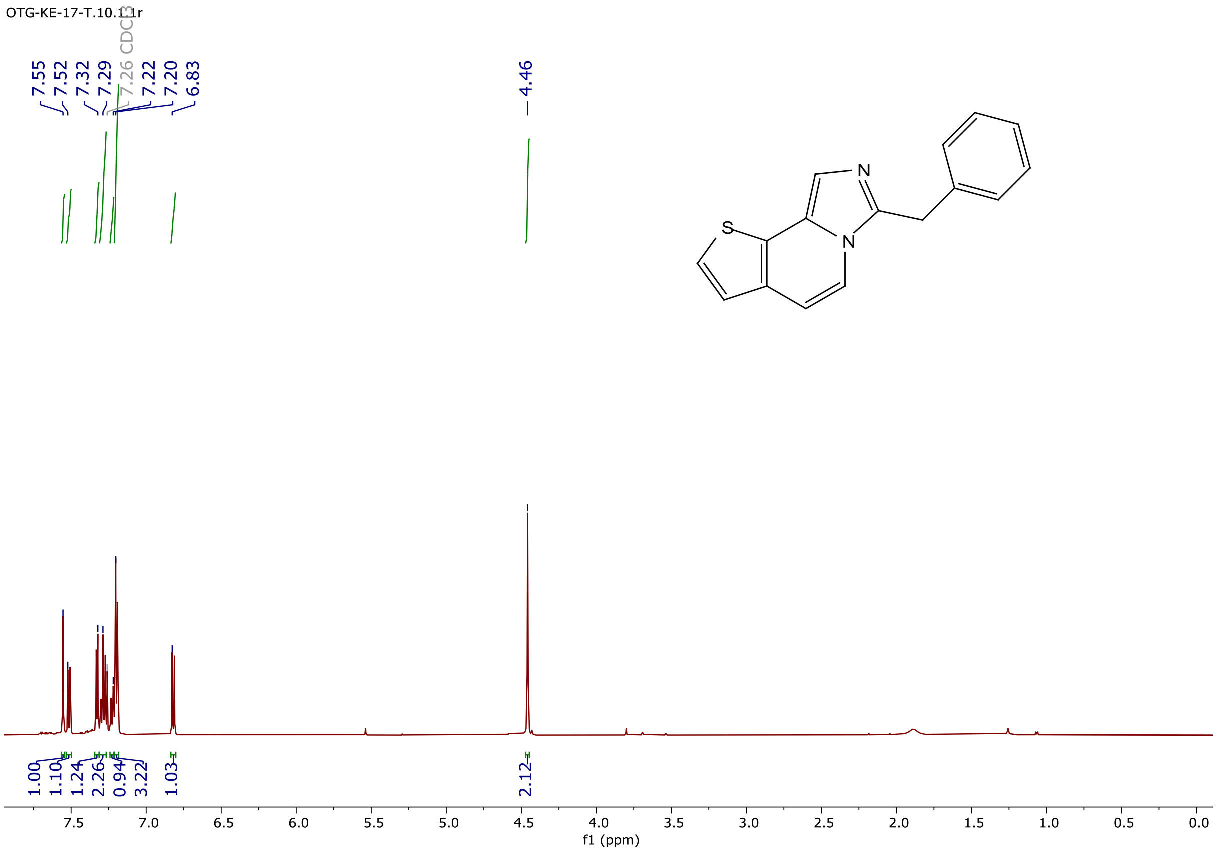

5b, ^{13}C (APT), 126 MHz, $CDCl_3$

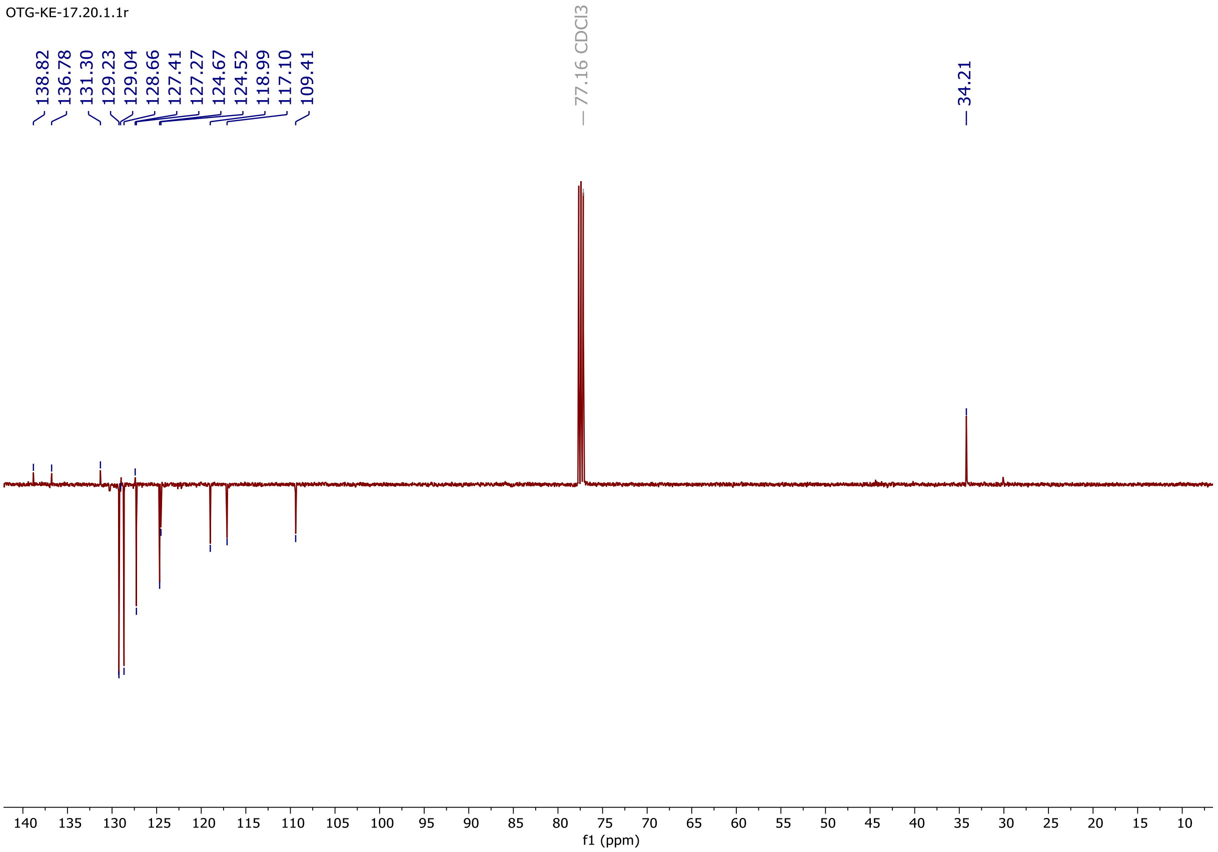

5c, ^{1}H, 500 MHz, $CDCl_3$

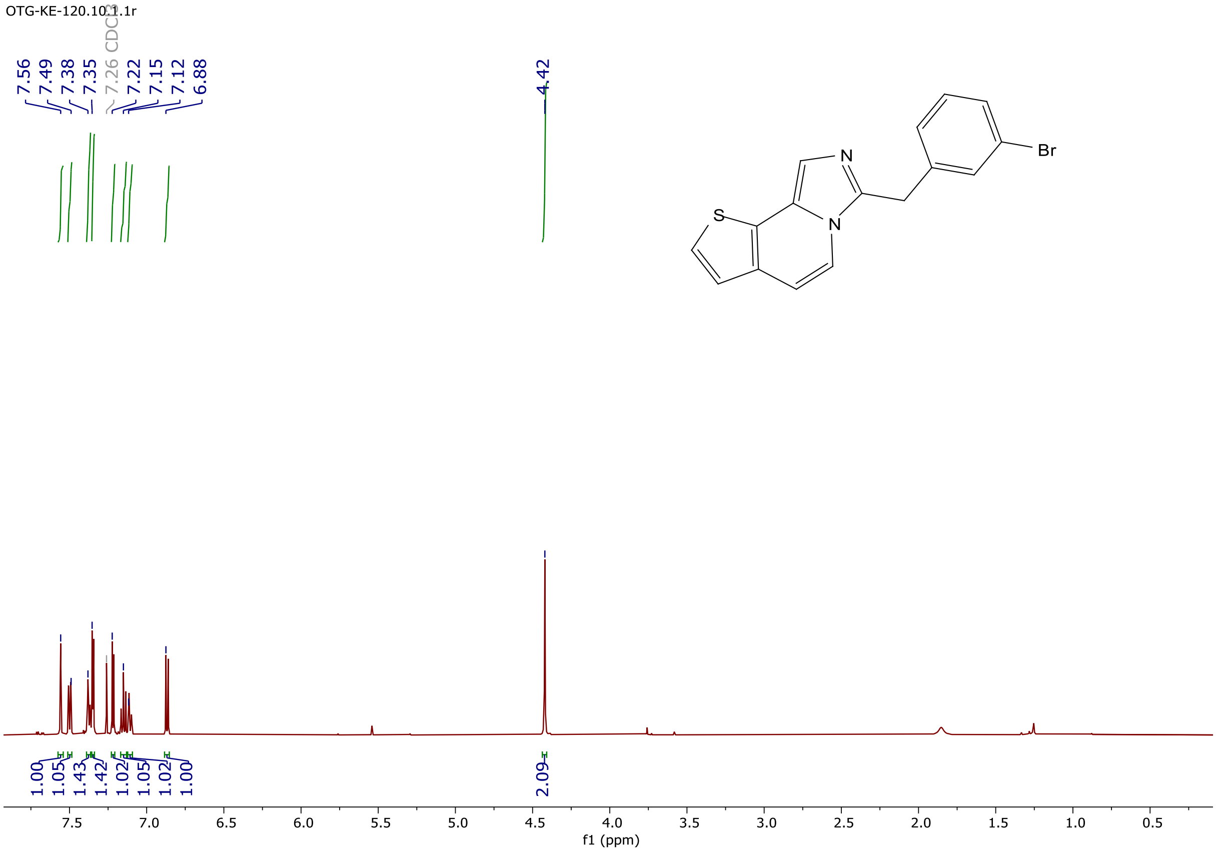

5c, ^{13}C (APT), 126 MHz, $CDCl_3$

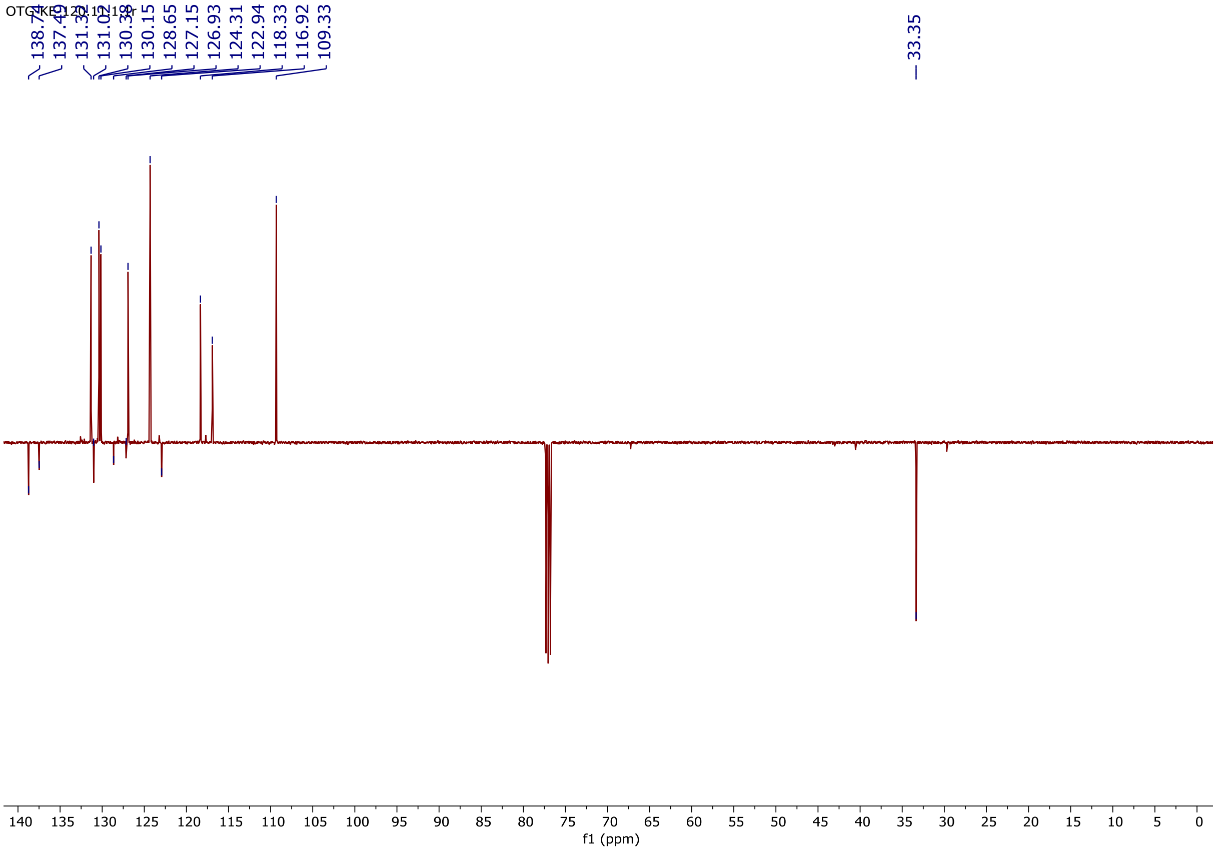

Supplement: Supplementary file 1 — Supporting Information [file OPEN-14-e202500060-s001.pdf]
